# Supplementary figures and images for: CYLD-TRAF6 interaction promotes ADP-heptose-induced NF-κB signaling in H. pylori infection
Source: EMBO Rep. 2025 May 22;26(13):3241–63. doi: 10.1038/s44319-025-00480-y (PMC12238516; doi:10.1038/s44319-025-00480-y)

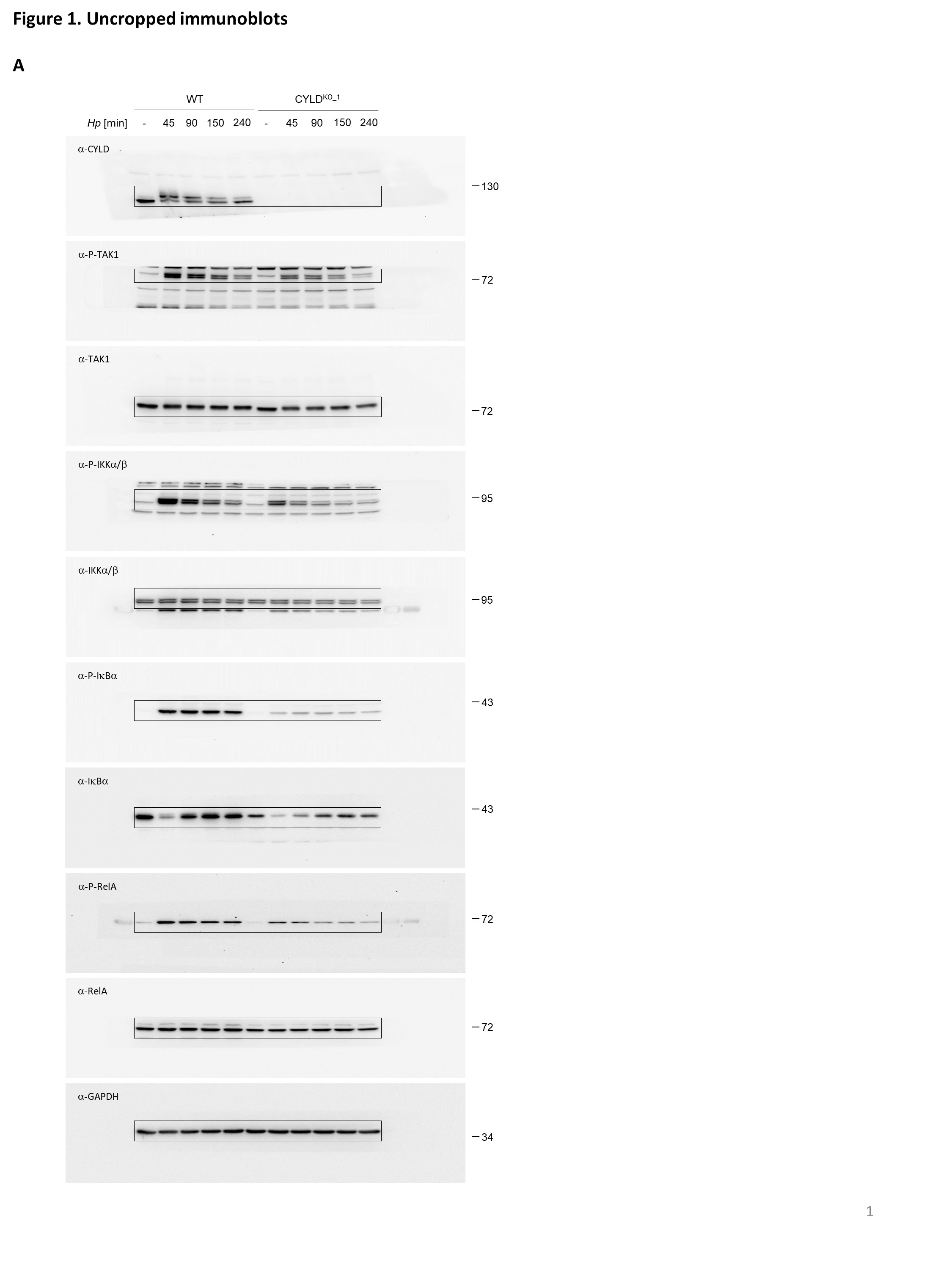

Supplement: Supplementary file 2 — Source data Fig. 1 [file 44319_2025_480_MOESM2_ESM.zip › Source data_Figure 1/Fig 1A.JPG]

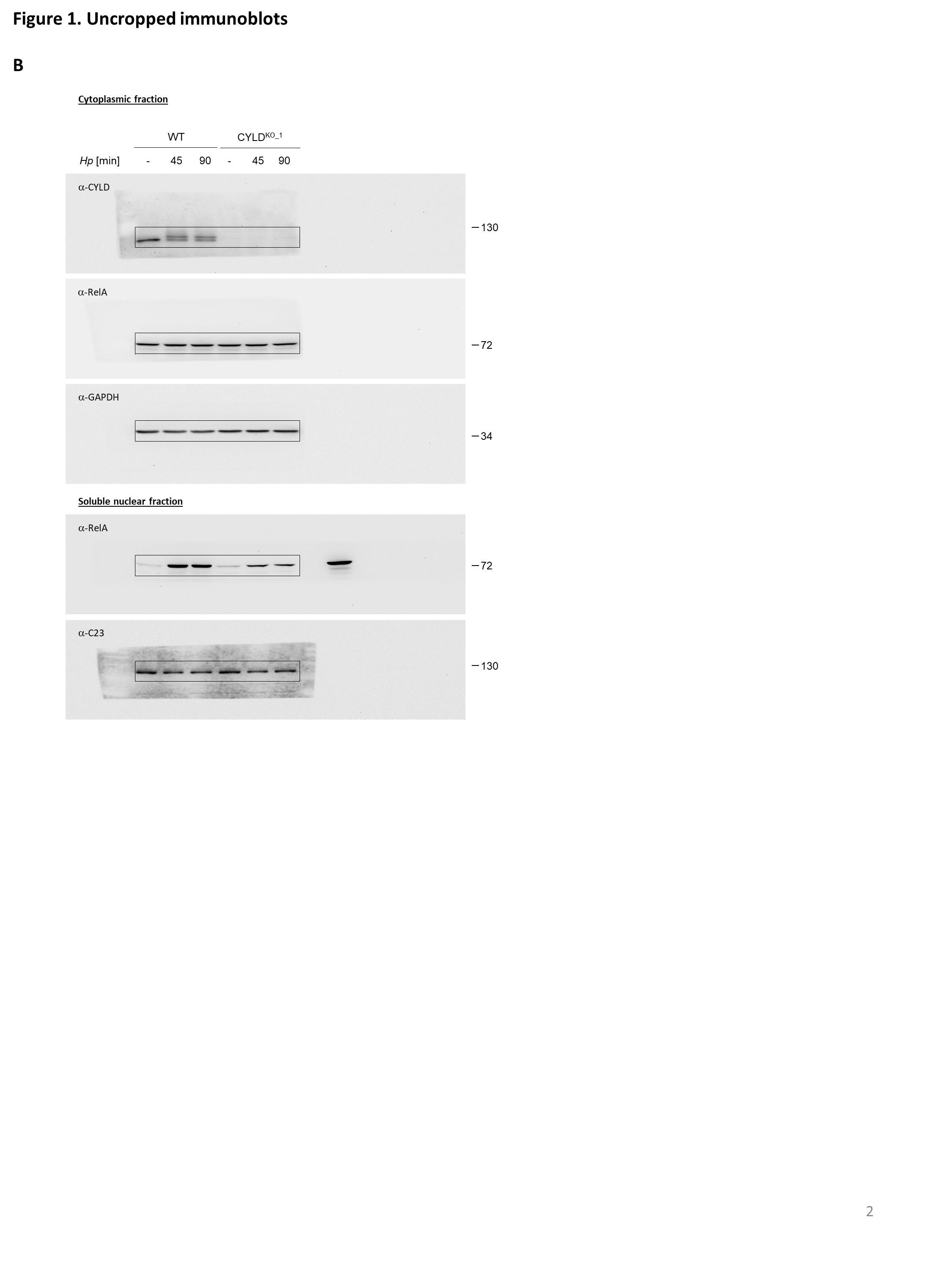

Supplement: Supplementary file 2 — Source data Fig. 1 [file 44319_2025_480_MOESM2_ESM.zip › Source data_Figure 1/Fig 1B.JPG]

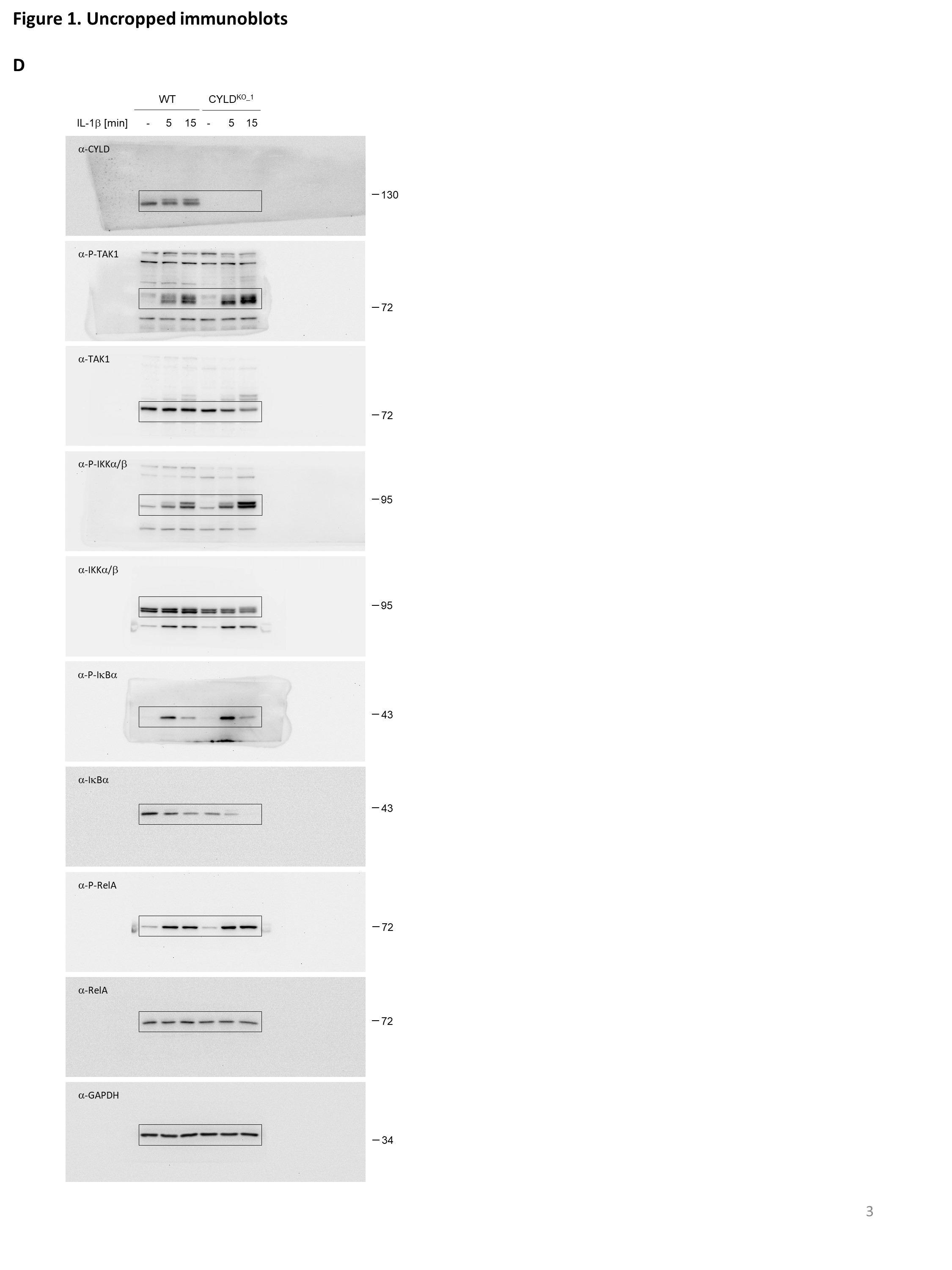

Supplement: Supplementary file 2 — Source data Fig. 1 [file 44319_2025_480_MOESM2_ESM.zip › Source data_Figure 1/Fig 1D.JPG]

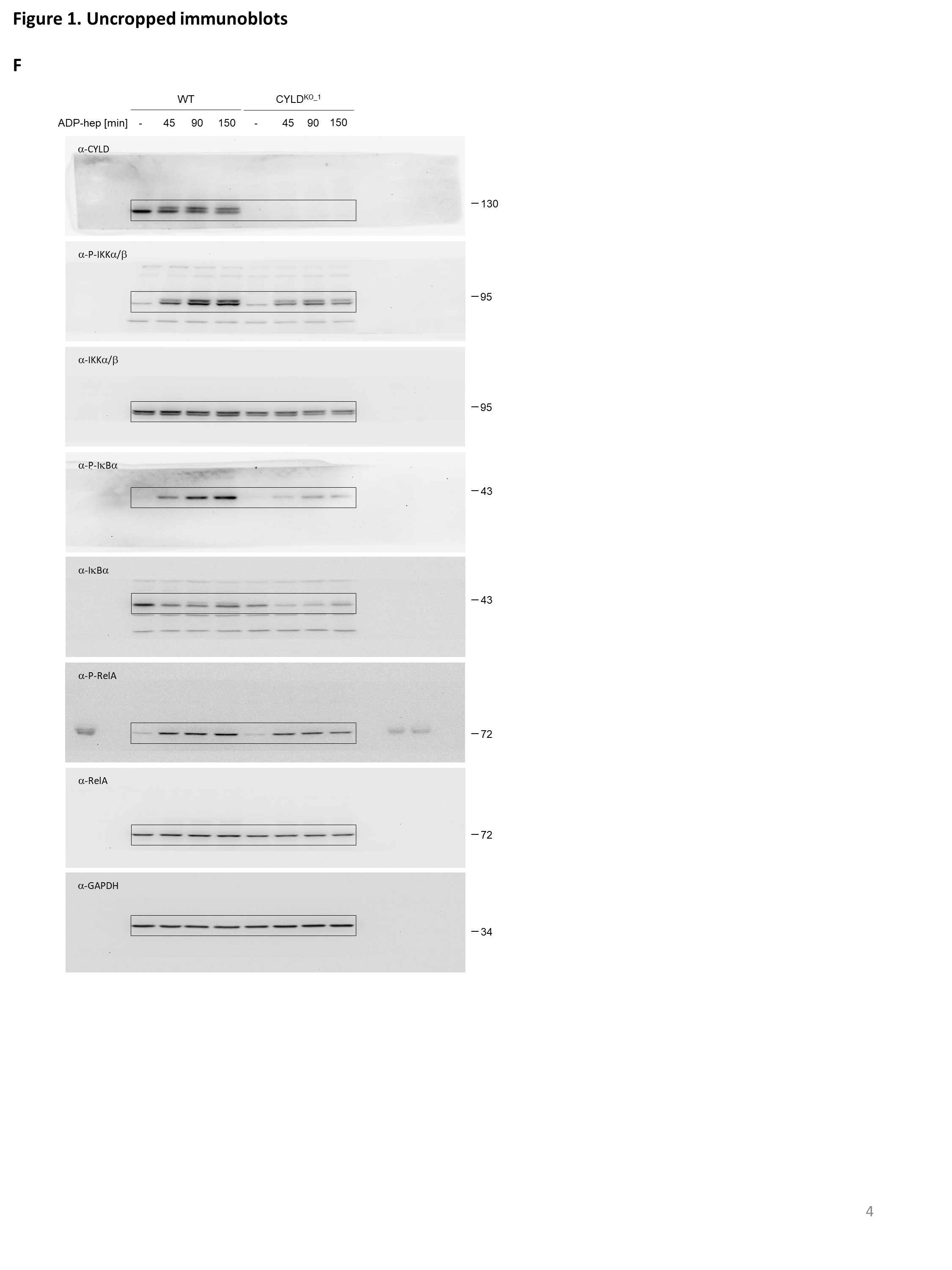

Supplement: Supplementary file 2 — Source data Fig. 1 [file 44319_2025_480_MOESM2_ESM.zip › Source data_Figure 1/Fig 1F.JPG]

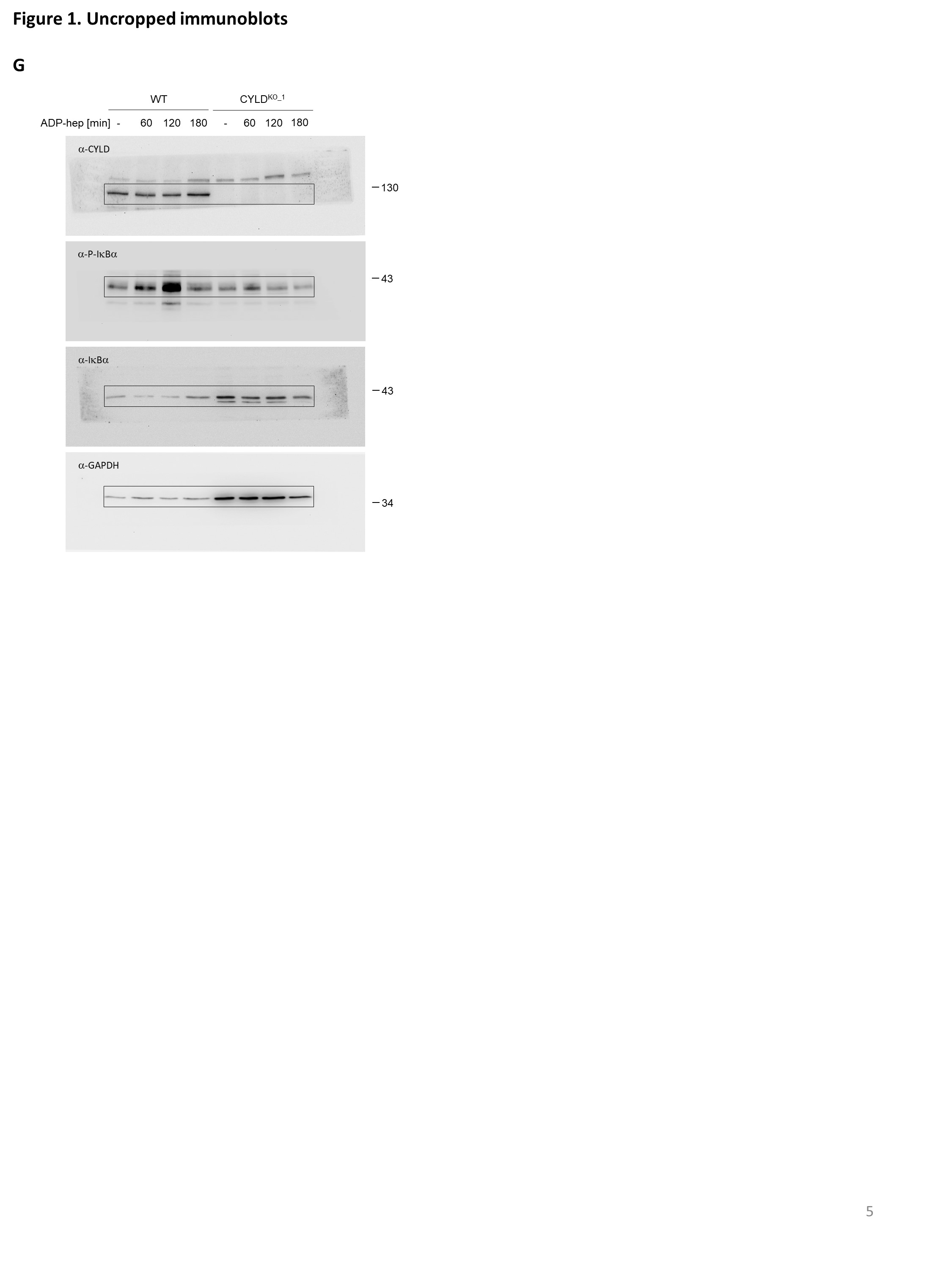

Supplement: Supplementary file 2 — Source data Fig. 1 [file 44319_2025_480_MOESM2_ESM.zip › Source data_Figure 1/Fig 1G.JPG]

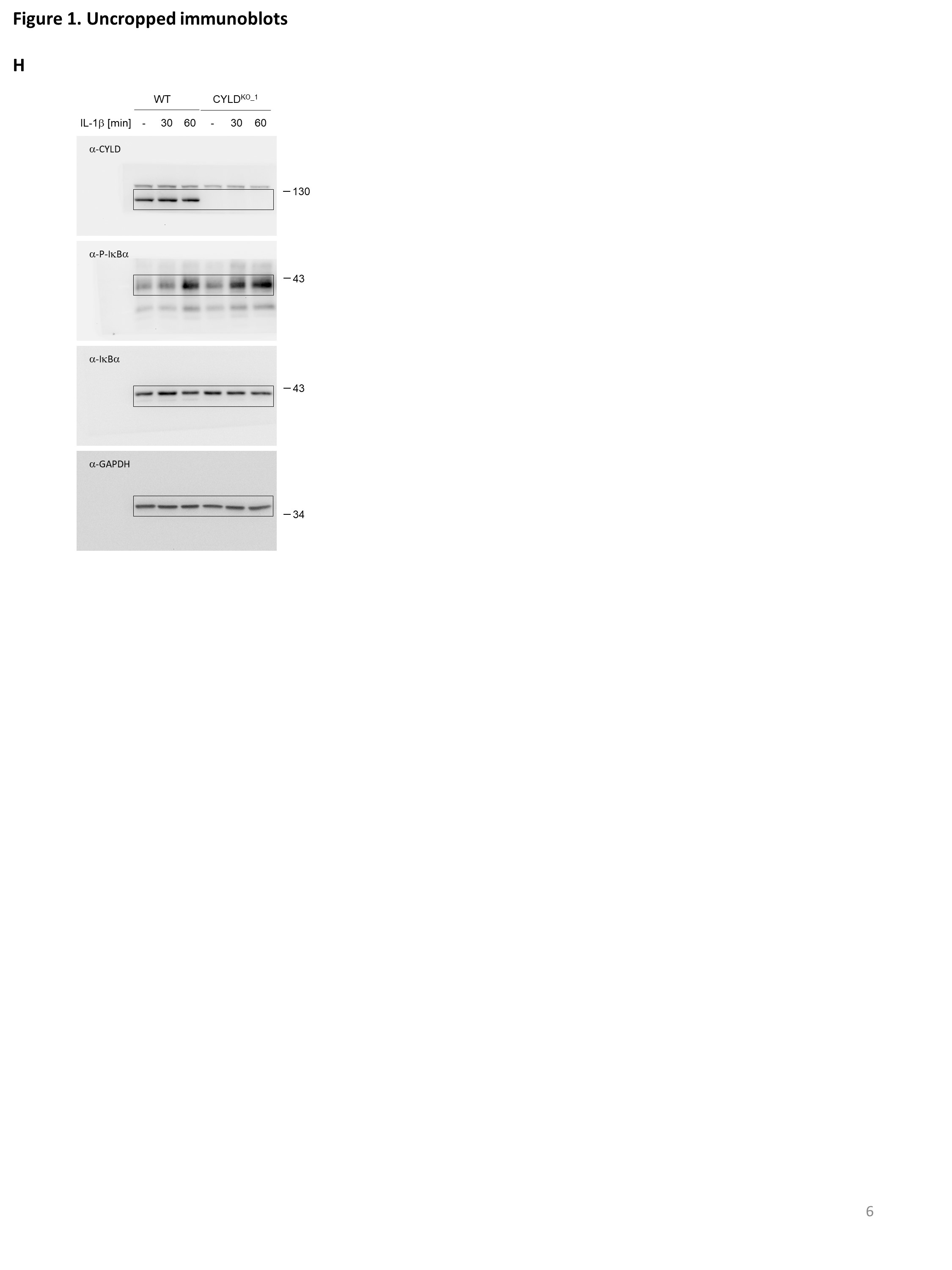

Supplement: Supplementary file 2 — Source data Fig. 1 [file 44319_2025_480_MOESM2_ESM.zip › Source data_Figure 1/Fig 1H.JPG]

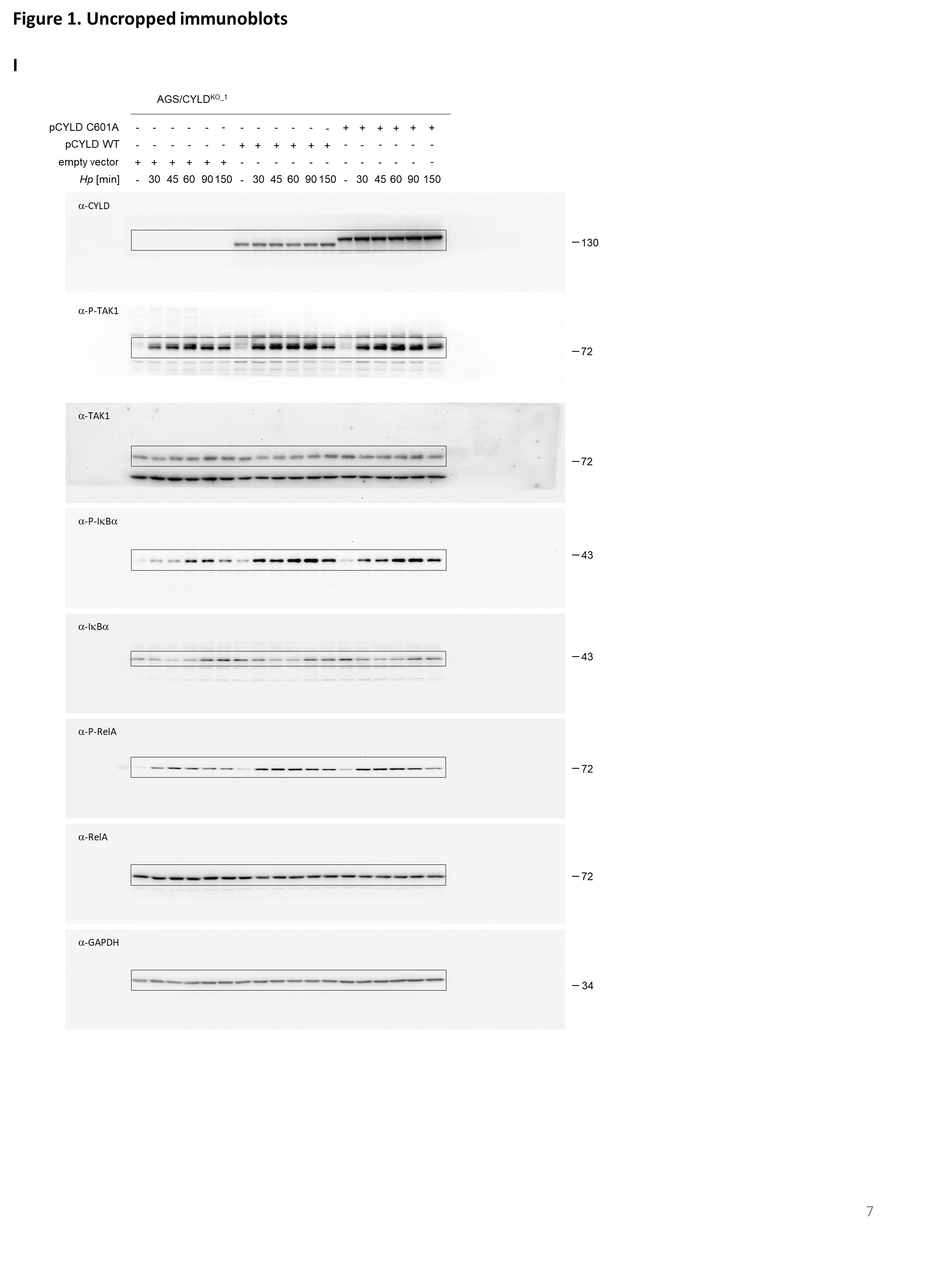

Supplement: Supplementary file 2 — Source data Fig. 1 [file 44319_2025_480_MOESM2_ESM.zip › Source data_Figure 1/Fig 1I.JPG]

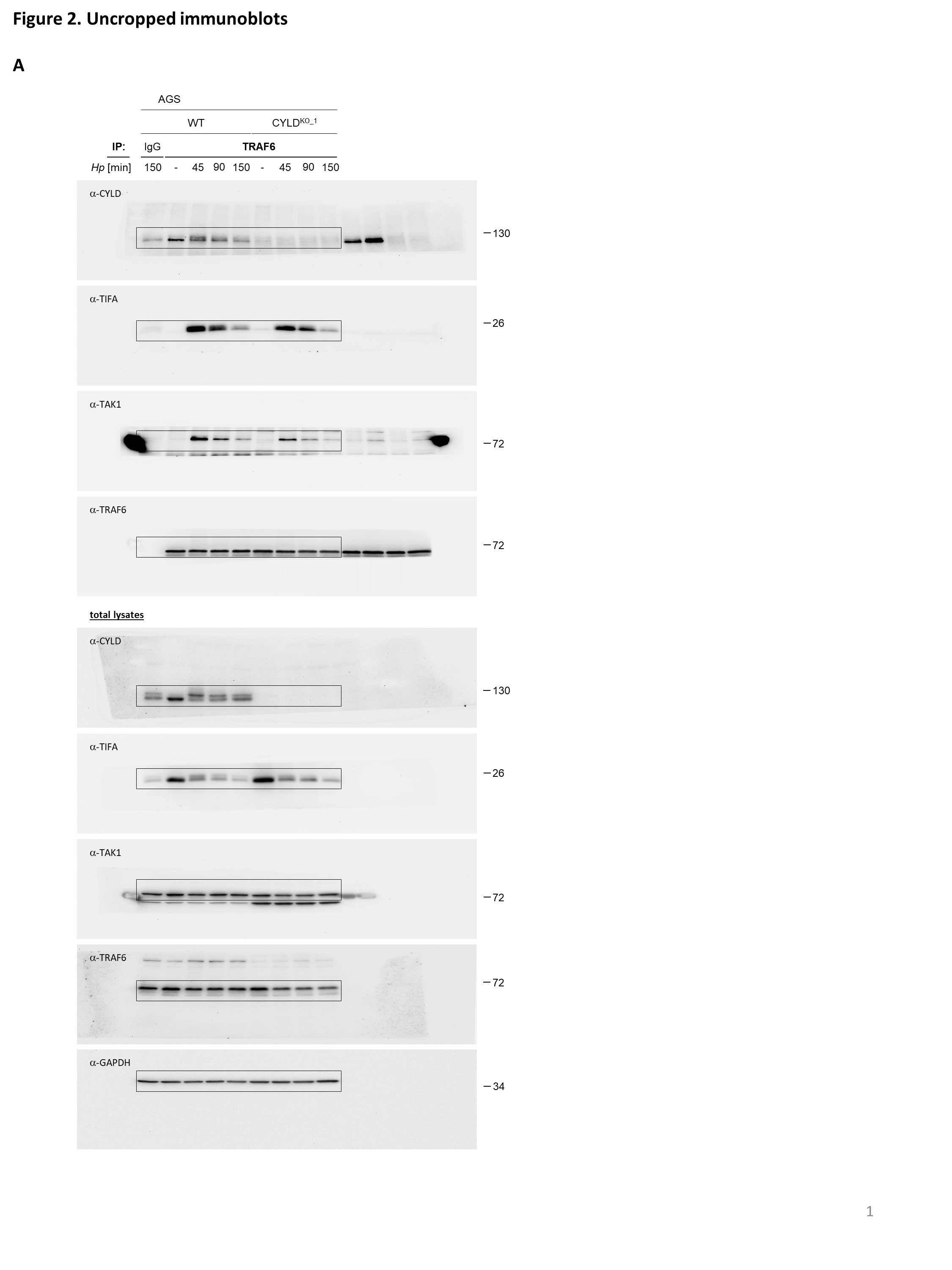

Supplement: Supplementary file 3 — Source data Fig. 2 [file 44319_2025_480_MOESM3_ESM.zip › Source data_Figure 2/Fig 2A.JPG]

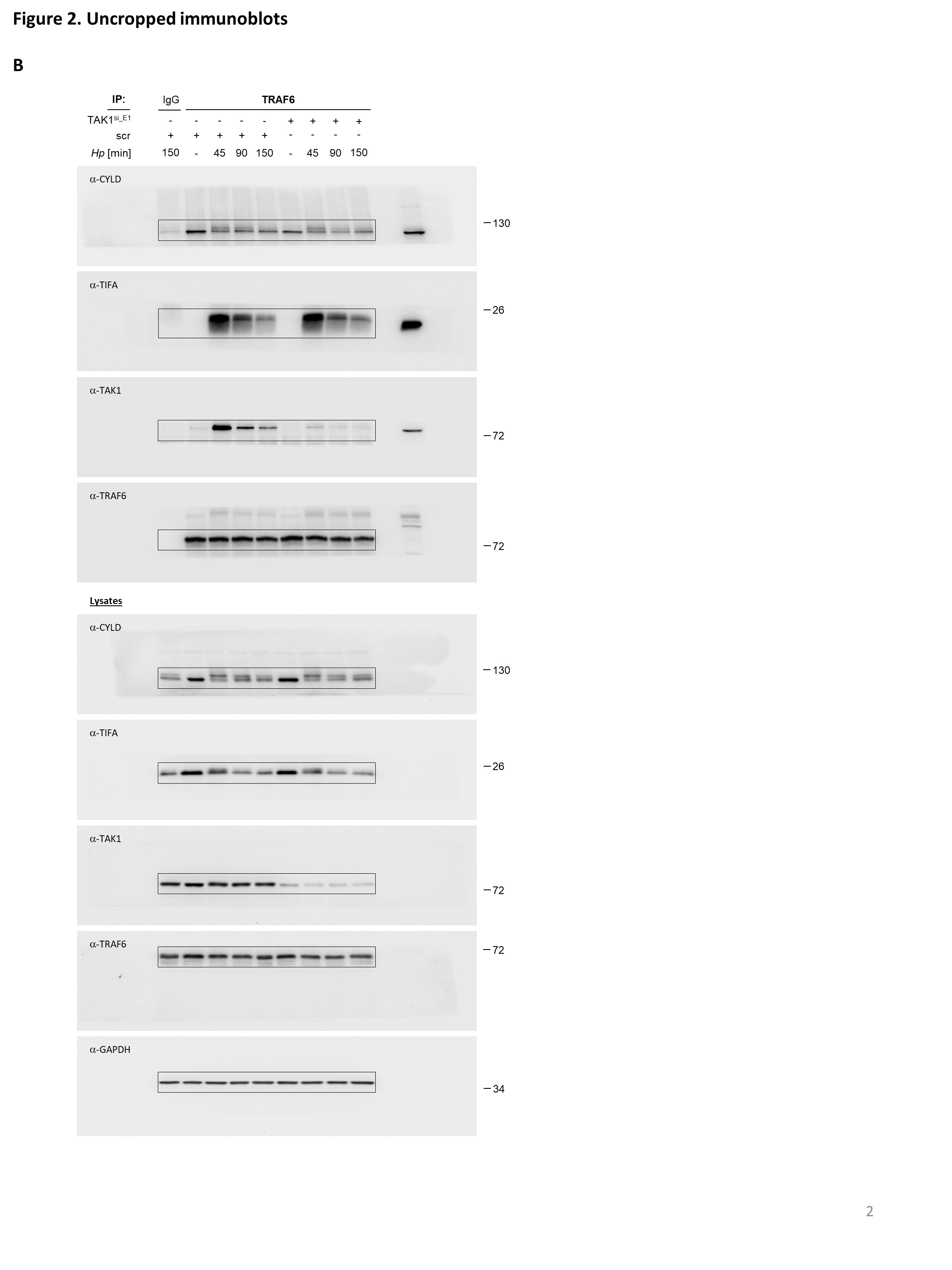

Supplement: Supplementary file 3 — Source data Fig. 2 [file 44319_2025_480_MOESM3_ESM.zip › Source data_Figure 2/Fig 2B.JPG]

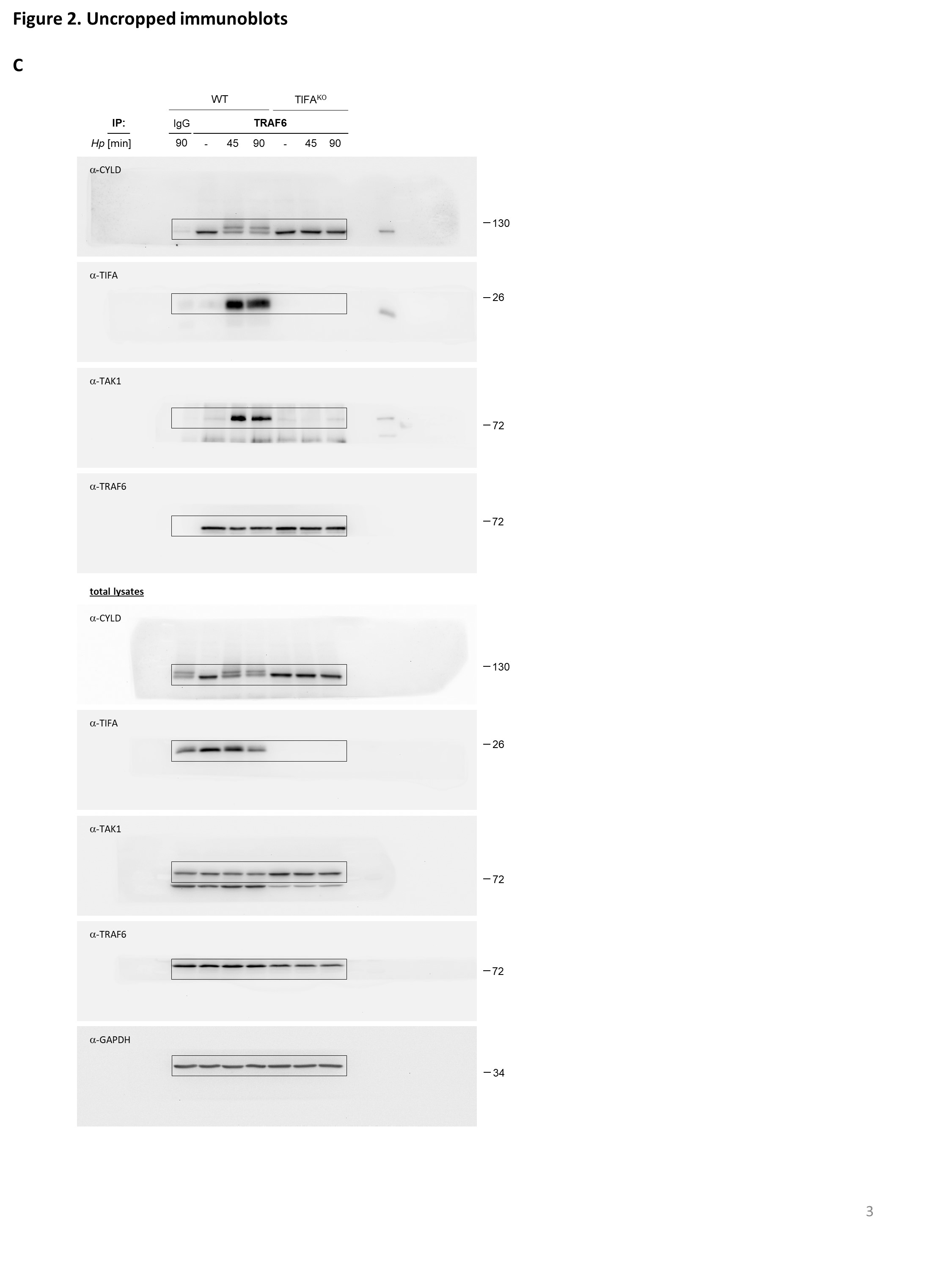

Supplement: Supplementary file 3 — Source data Fig. 2 [file 44319_2025_480_MOESM3_ESM.zip › Source data_Figure 2/Fig 2C.JPG]

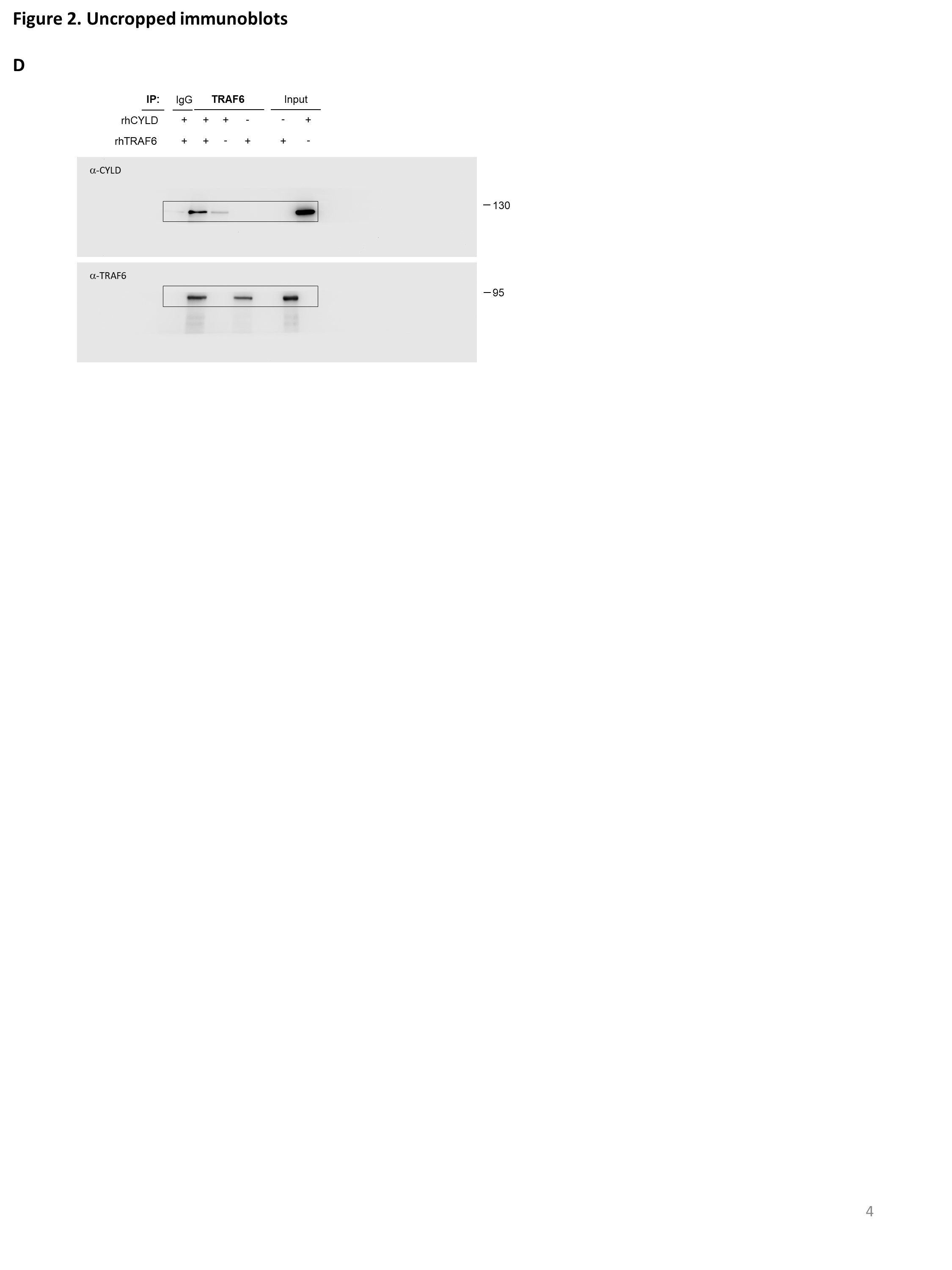

Supplement: Supplementary file 3 — Source data Fig. 2 [file 44319_2025_480_MOESM3_ESM.zip › Source data_Figure 2/Fig 2D.JPG]

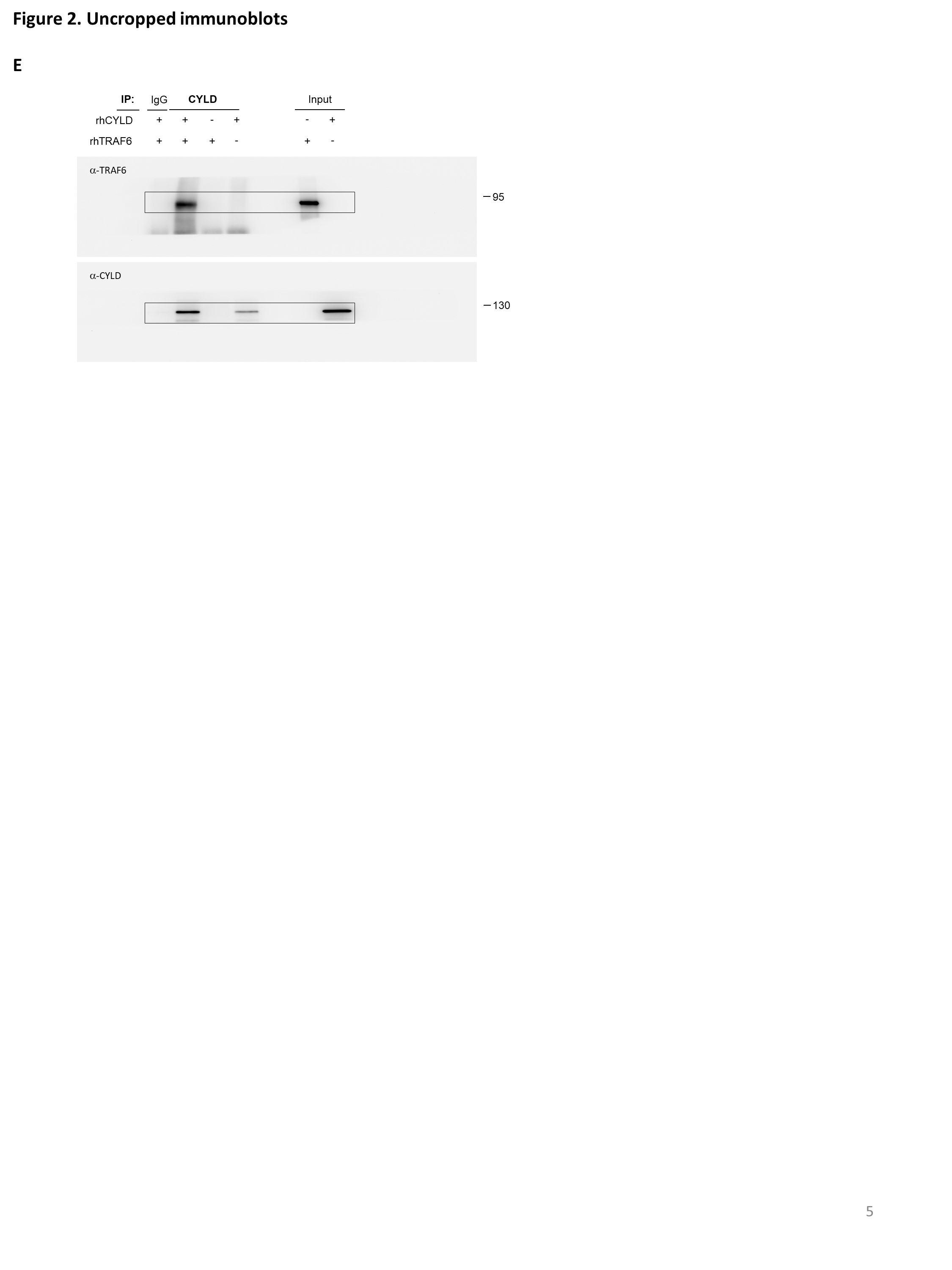

Supplement: Supplementary file 3 — Source data Fig. 2 [file 44319_2025_480_MOESM3_ESM.zip › Source data_Figure 2/Fig 2E.JPG]

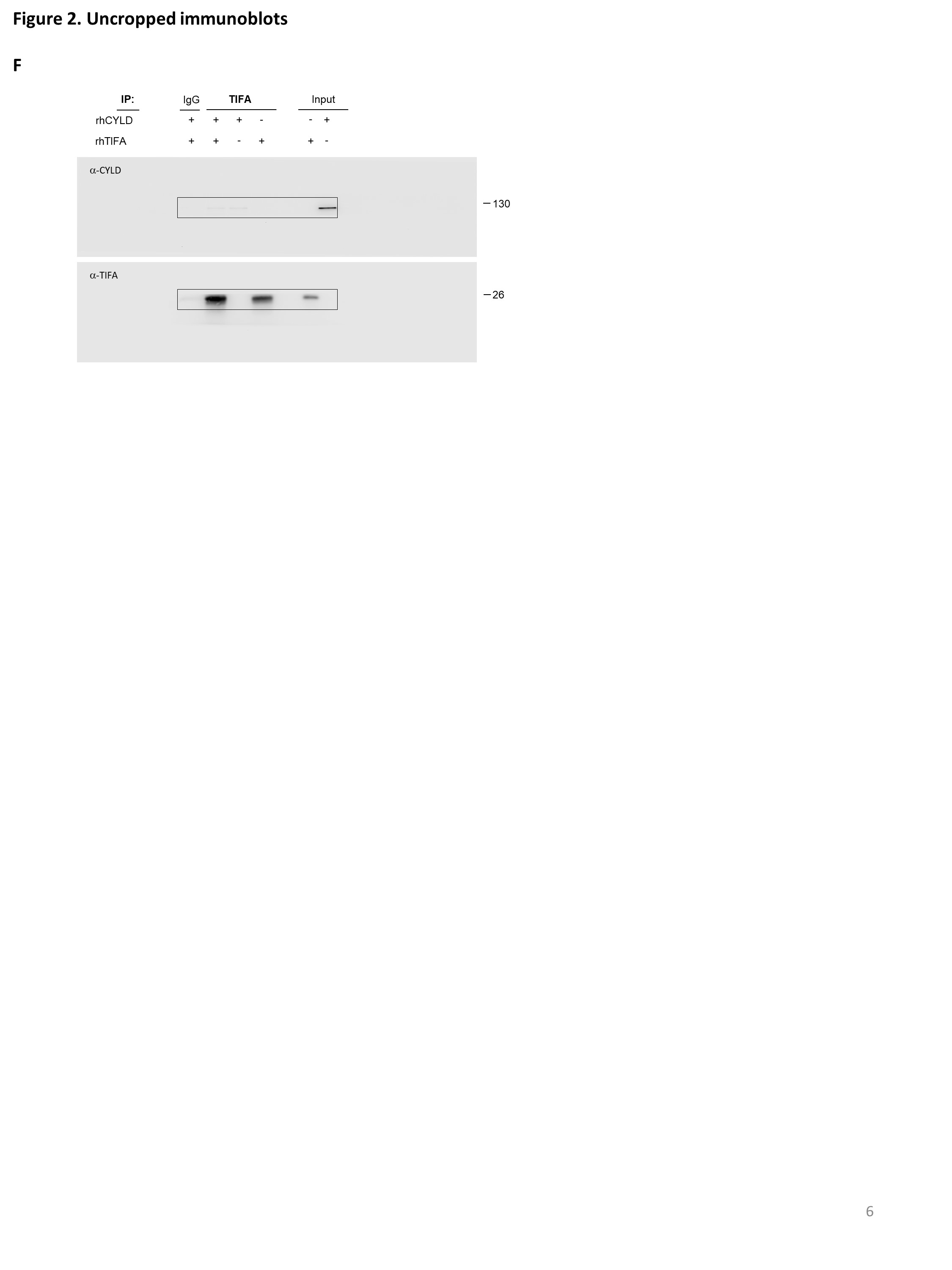

Supplement: Supplementary file 3 — Source data Fig. 2 [file 44319_2025_480_MOESM3_ESM.zip › Source data_Figure 2/Fig 2F.JPG]

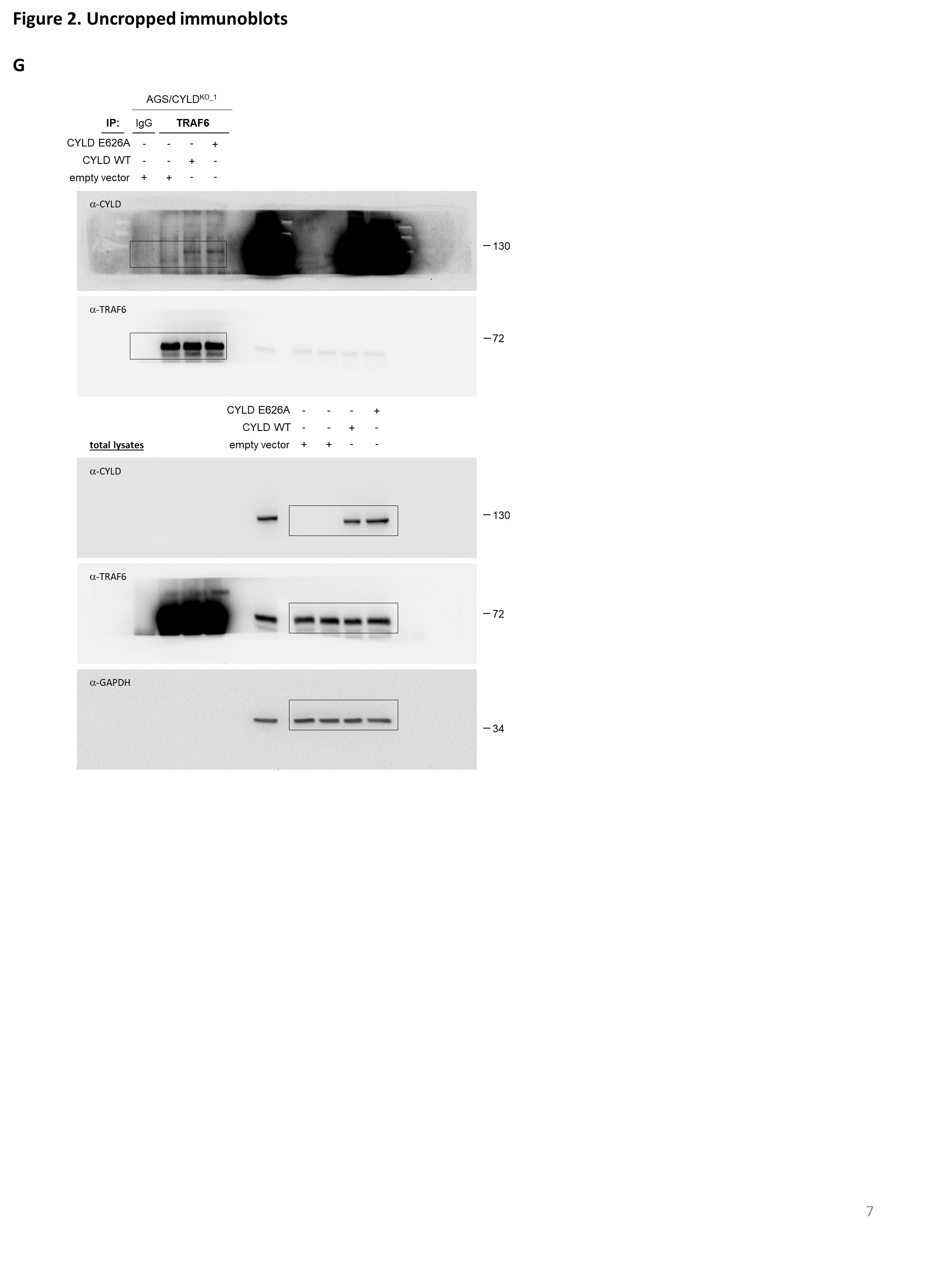

Supplement: Supplementary file 3 — Source data Fig. 2 [file 44319_2025_480_MOESM3_ESM.zip › Source data_Figure 2/Fig 2G.JPG]

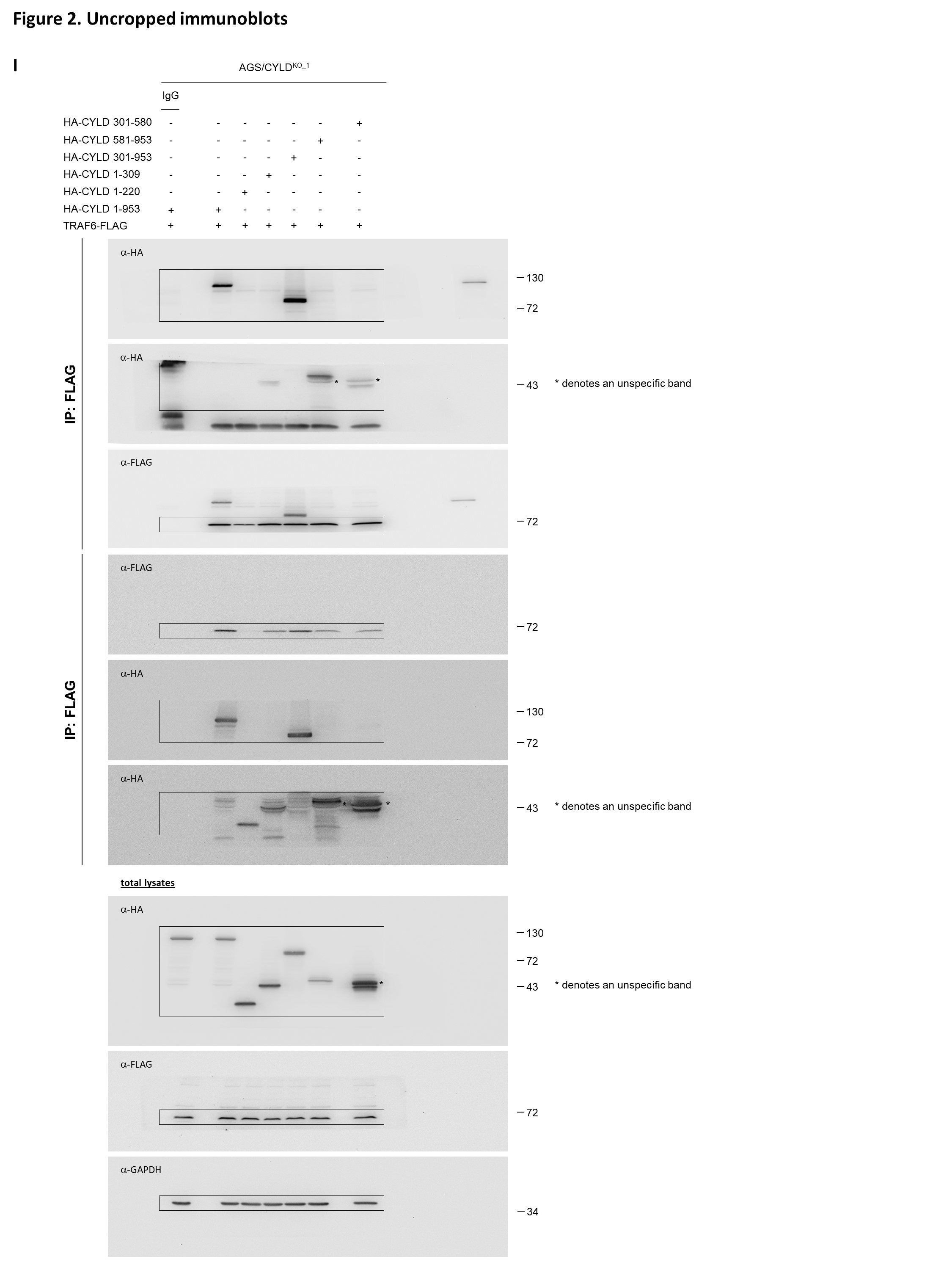

Supplement: Supplementary file 3 — Source data Fig. 2 [file 44319_2025_480_MOESM3_ESM.zip › Source data_Figure 2/Fig 2I.JPG]

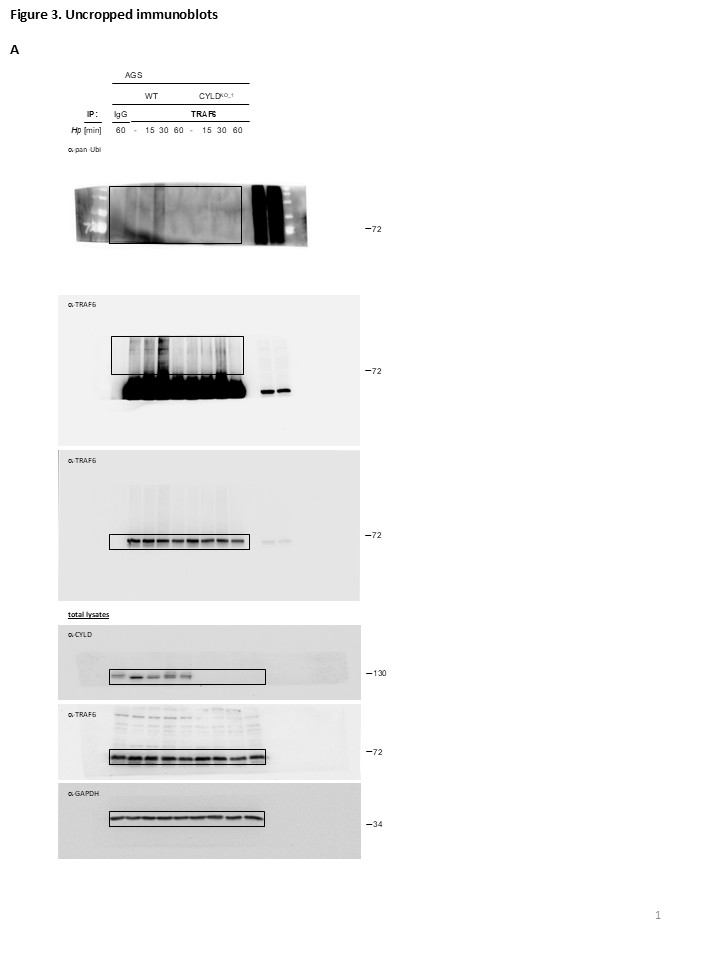

Supplement: Supplementary file 4 — Source data Fig. 3 [file 44319_2025_480_MOESM4_ESM.zip › Source data_Figure 3/Fig 3A.JPG]

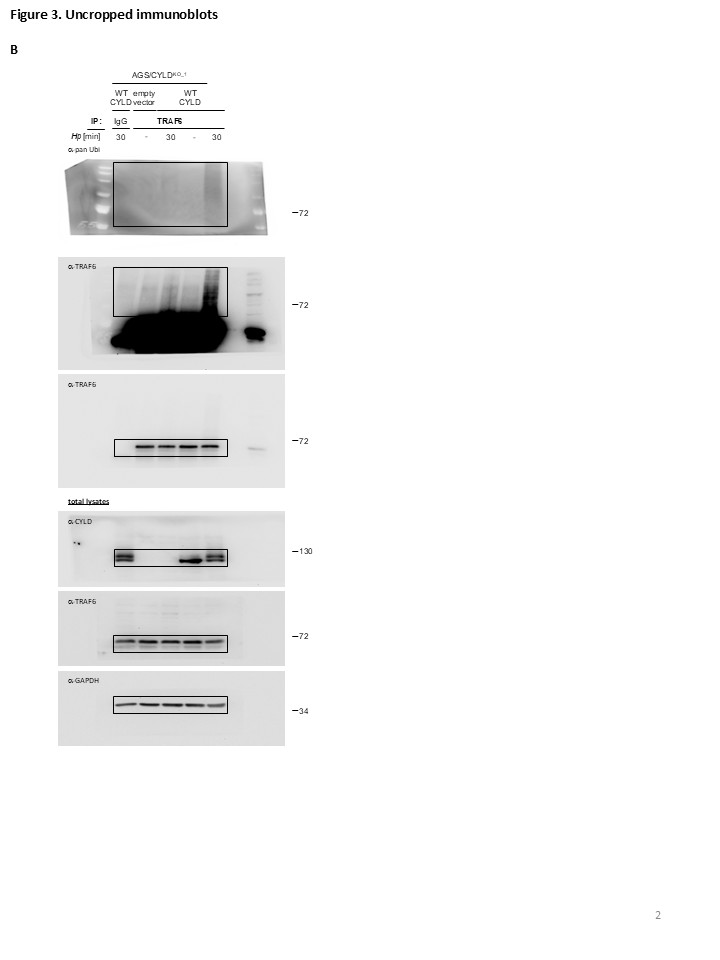

Supplement: Supplementary file 4 — Source data Fig. 3 [file 44319_2025_480_MOESM4_ESM.zip › Source data_Figure 3/Fig 3B.JPG]

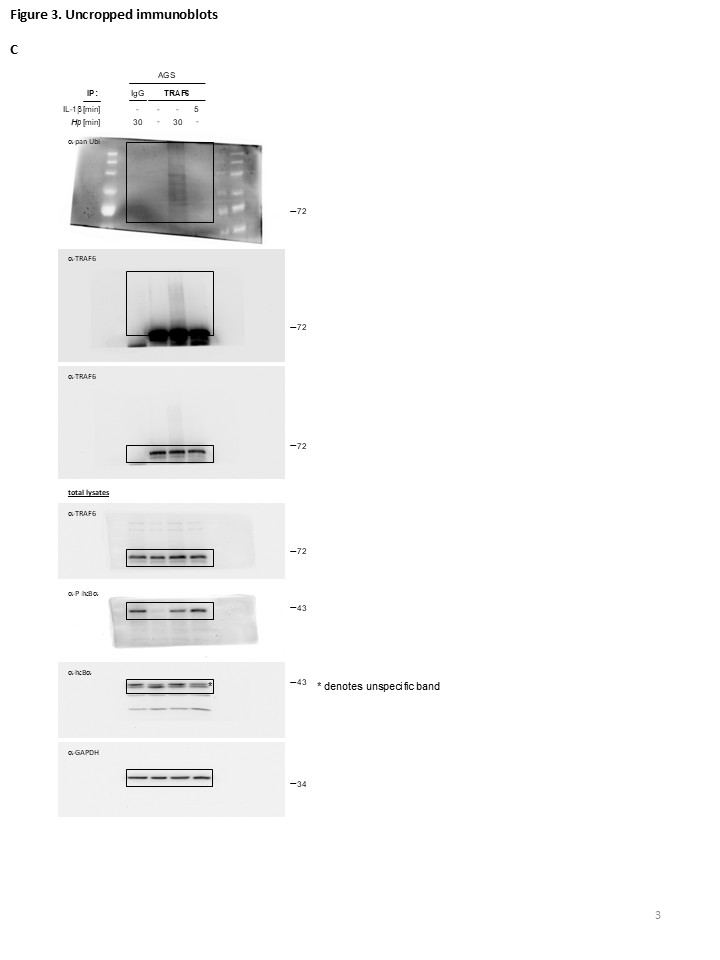

Supplement: Supplementary file 4 — Source data Fig. 3 [file 44319_2025_480_MOESM4_ESM.zip › Source data_Figure 3/Fig 3C.JPG]

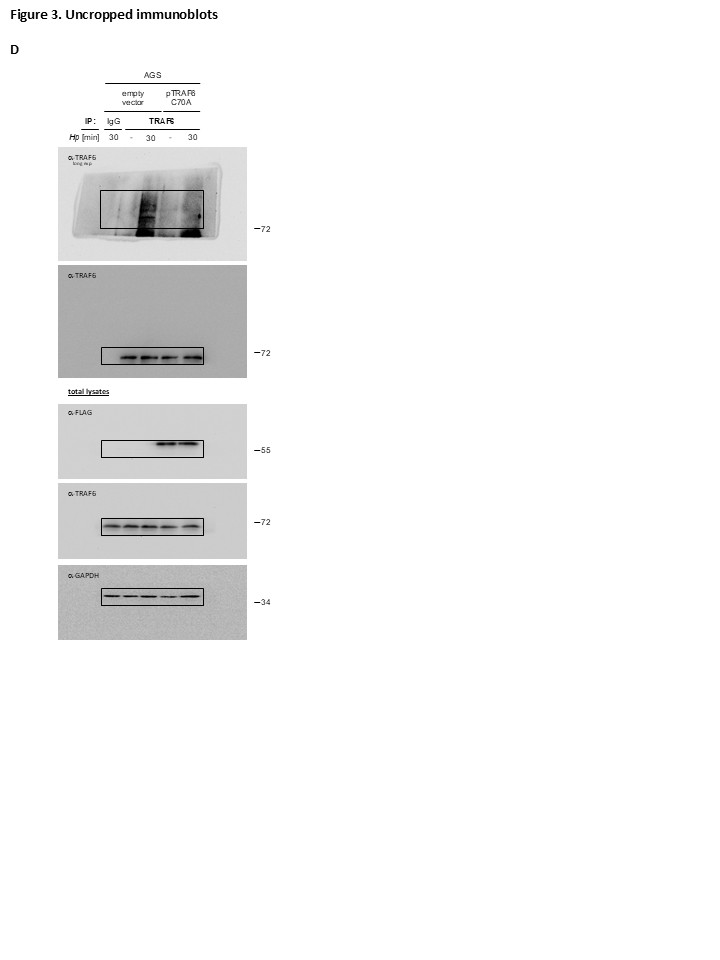

Supplement: Supplementary file 4 — Source data Fig. 3 [file 44319_2025_480_MOESM4_ESM.zip › Source data_Figure 3/Fig 3D.JPG]

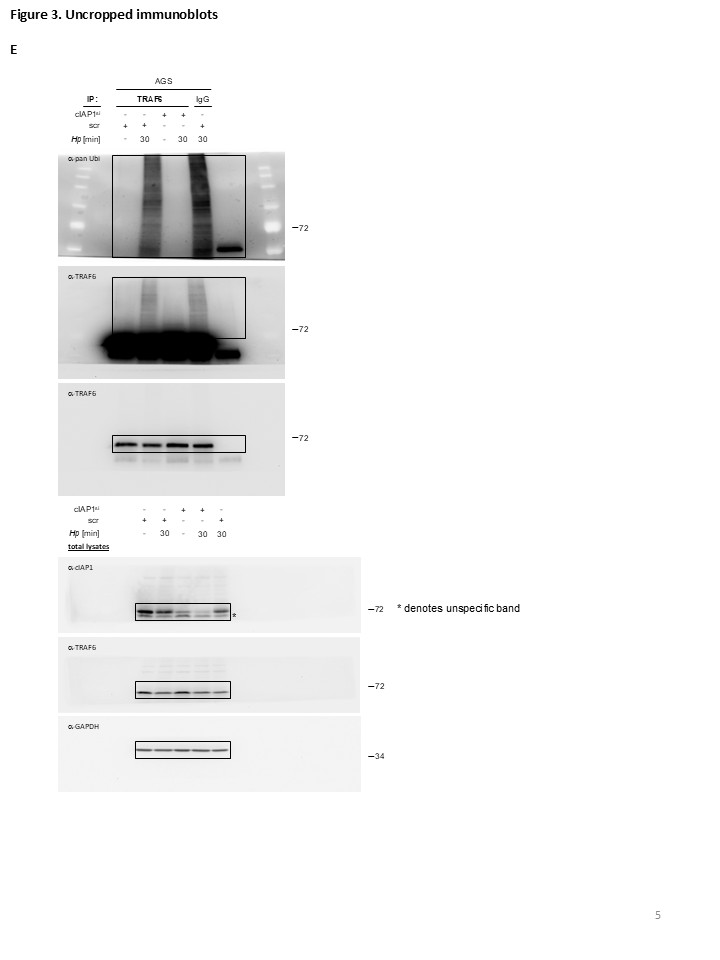

Supplement: Supplementary file 4 — Source data Fig. 3 [file 44319_2025_480_MOESM4_ESM.zip › Source data_Figure 3/Fig 3E.JPG]

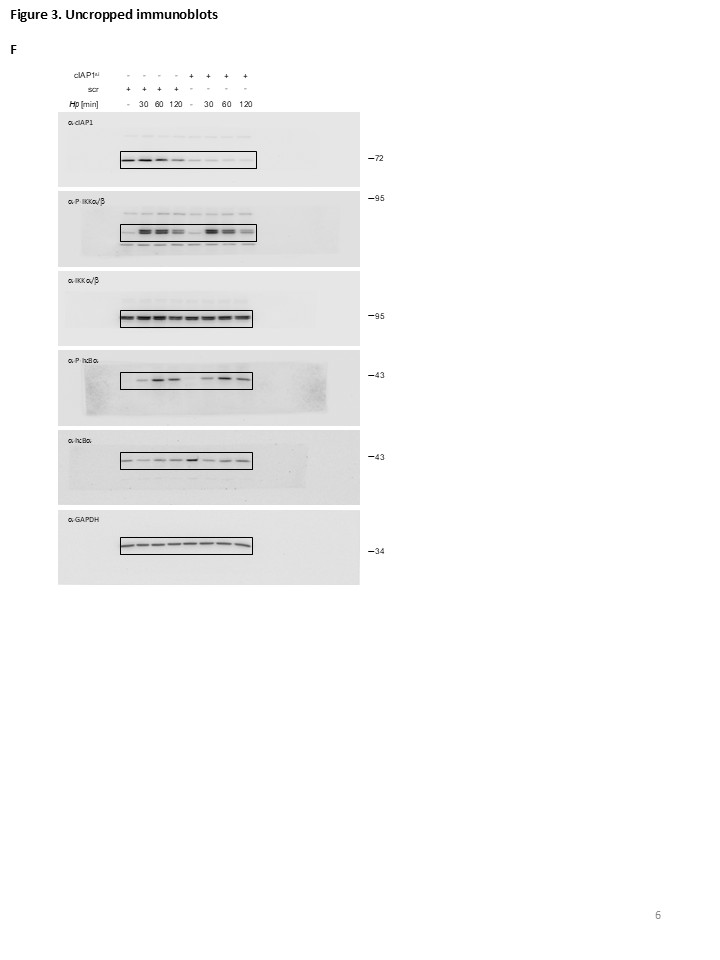

Supplement: Supplementary file 4 — Source data Fig. 3 [file 44319_2025_480_MOESM4_ESM.zip › Source data_Figure 3/Fig 3F.JPG]

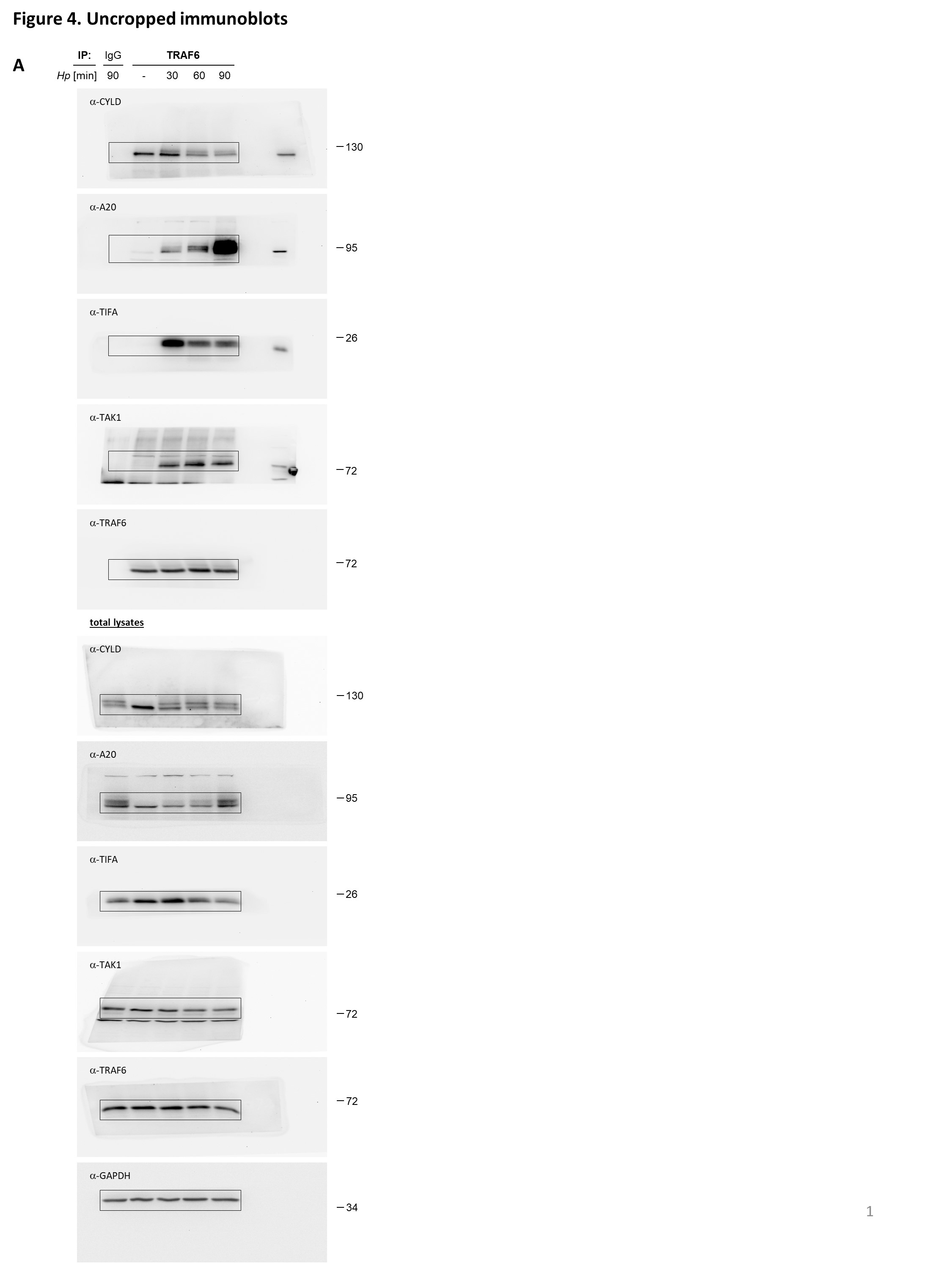

Supplement: Supplementary file 5 — Source data Fig. 4 [file 44319_2025_480_MOESM5_ESM.zip › Source data_Figure 4/Fig 4A.JPG]

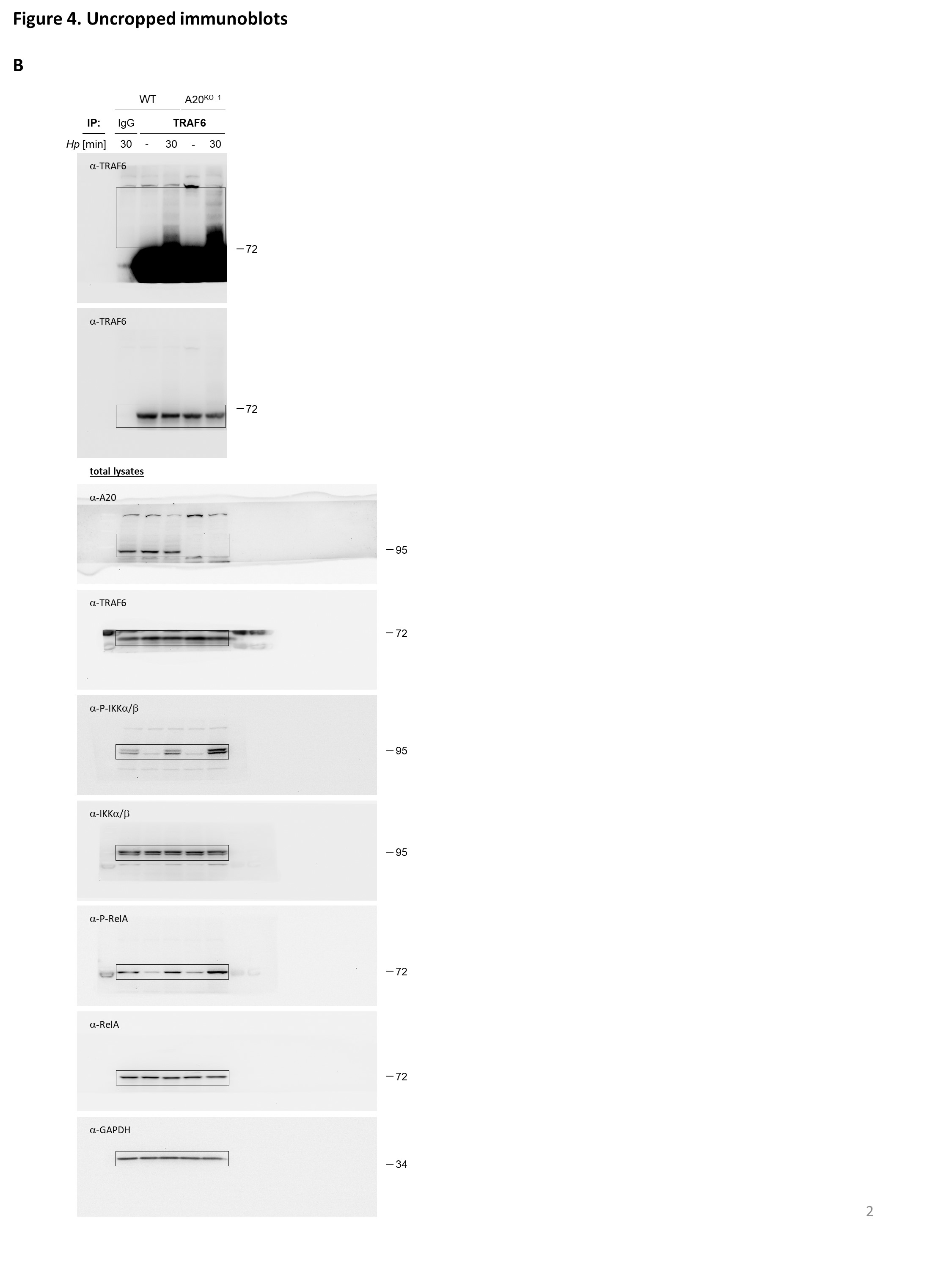

Supplement: Supplementary file 5 — Source data Fig. 4 [file 44319_2025_480_MOESM5_ESM.zip › Source data_Figure 4/Fig 4B.JPG]

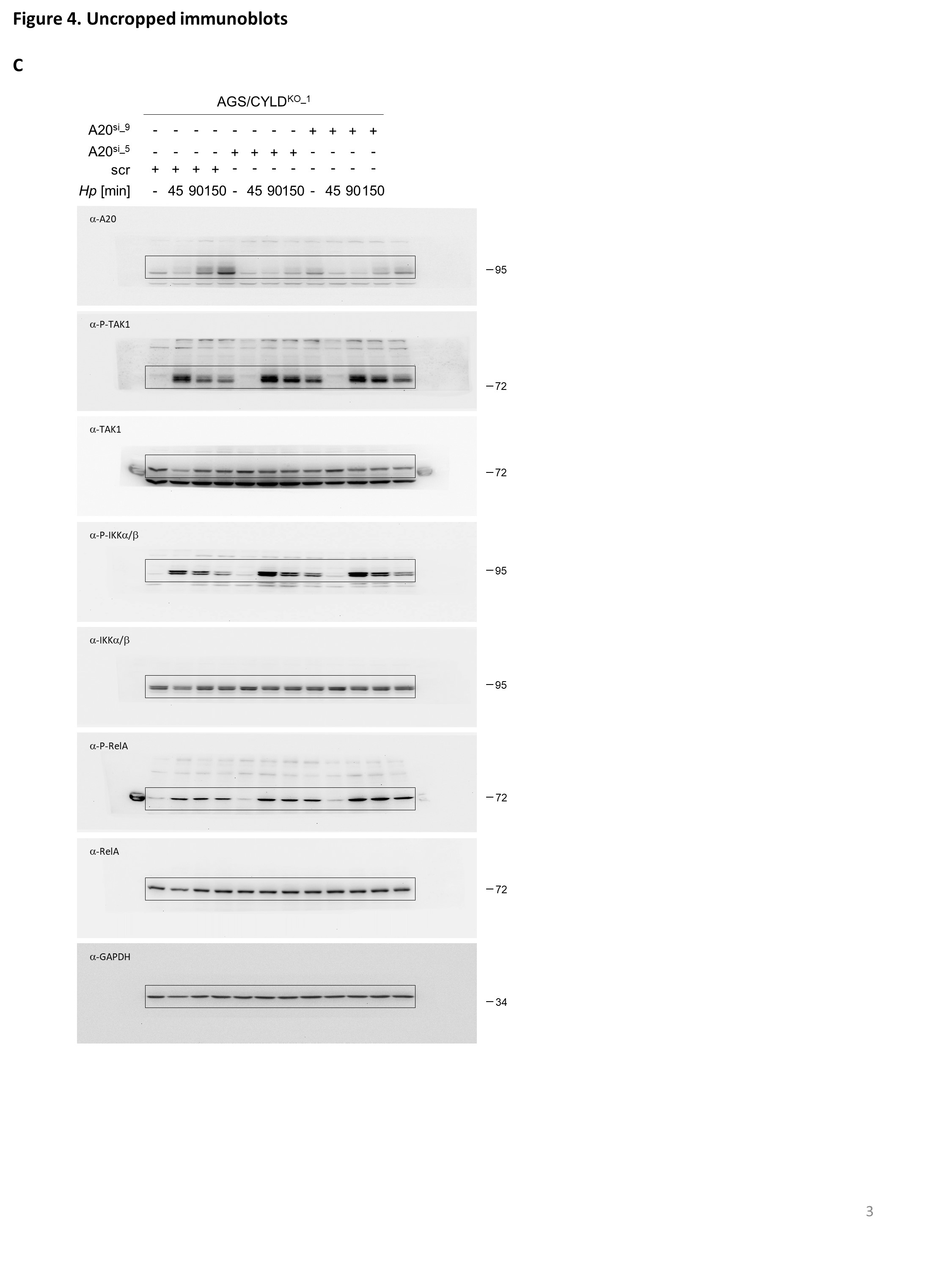

Supplement: Supplementary file 5 — Source data Fig. 4 [file 44319_2025_480_MOESM5_ESM.zip › Source data_Figure 4/Fig 4C.JPG]

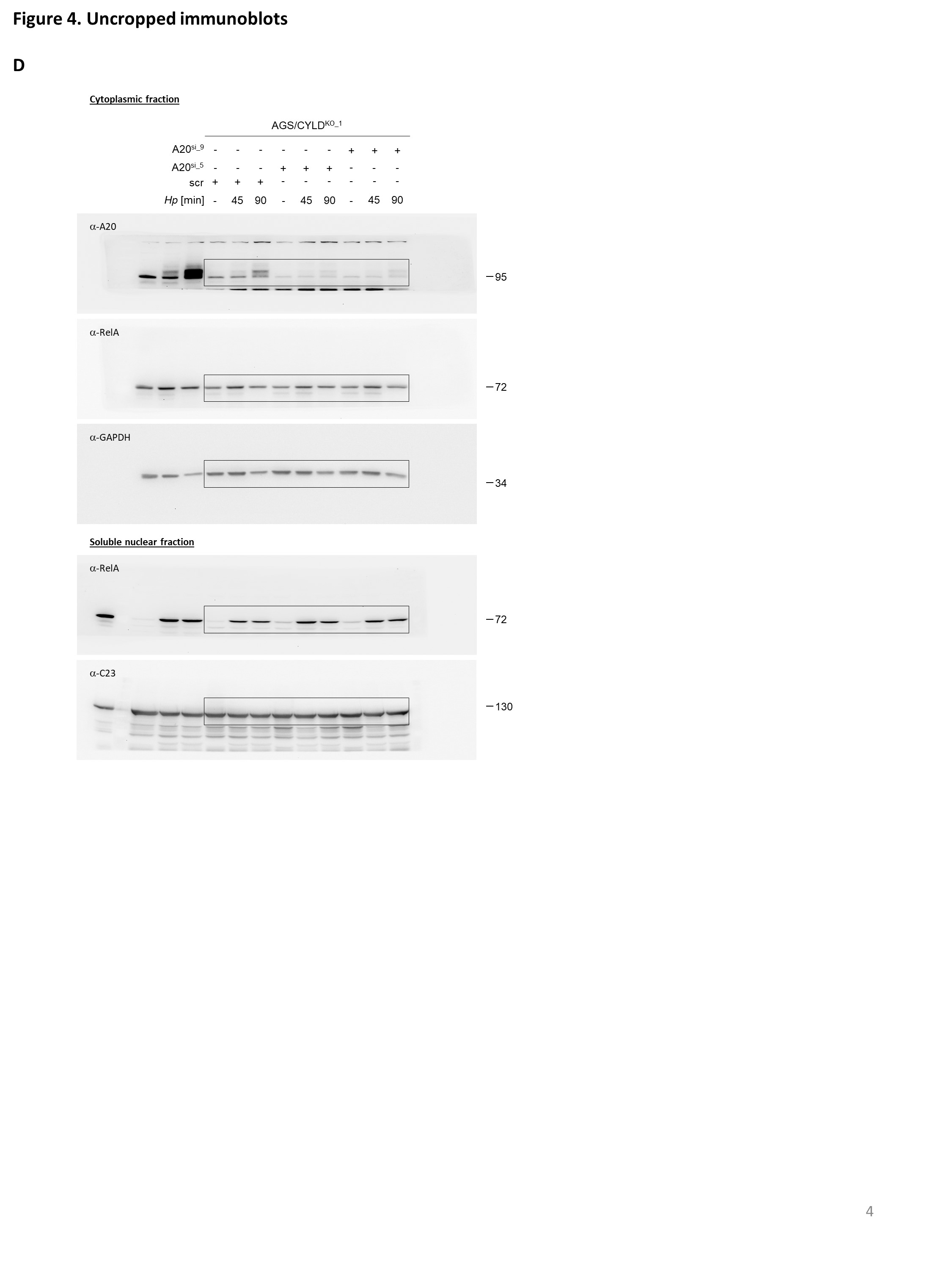

Supplement: Supplementary file 5 — Source data Fig. 4 [file 44319_2025_480_MOESM5_ESM.zip › Source data_Figure 4/Fig 4D.JPG]

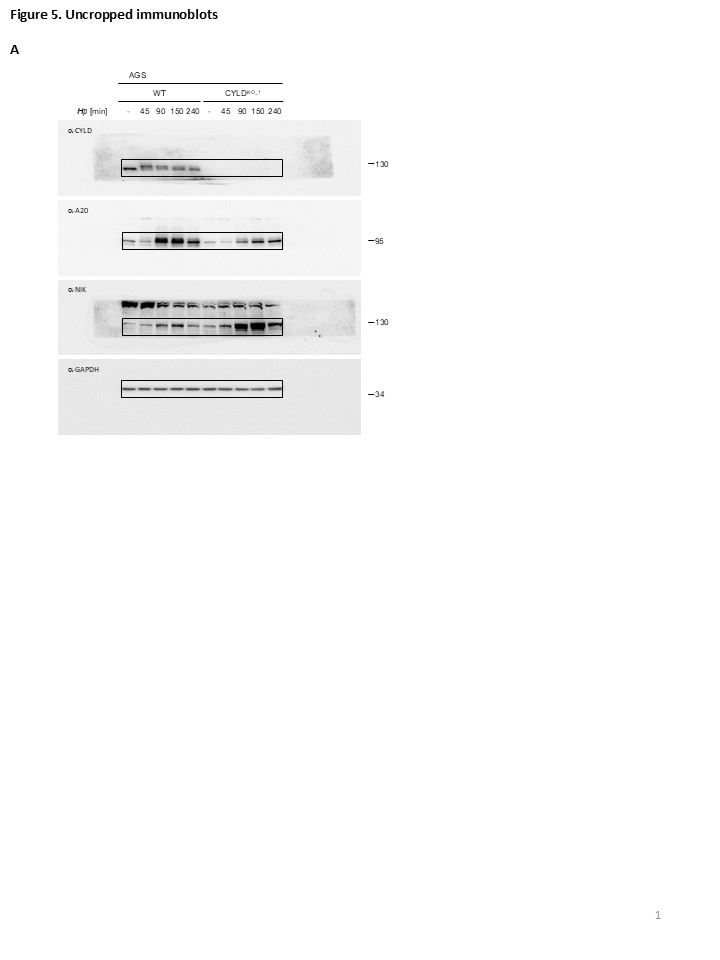

Supplement: Supplementary file 6 — Source data Fig. 5 [file 44319_2025_480_MOESM6_ESM.zip › Source data_Figure 5/Fig 5A.JPG]

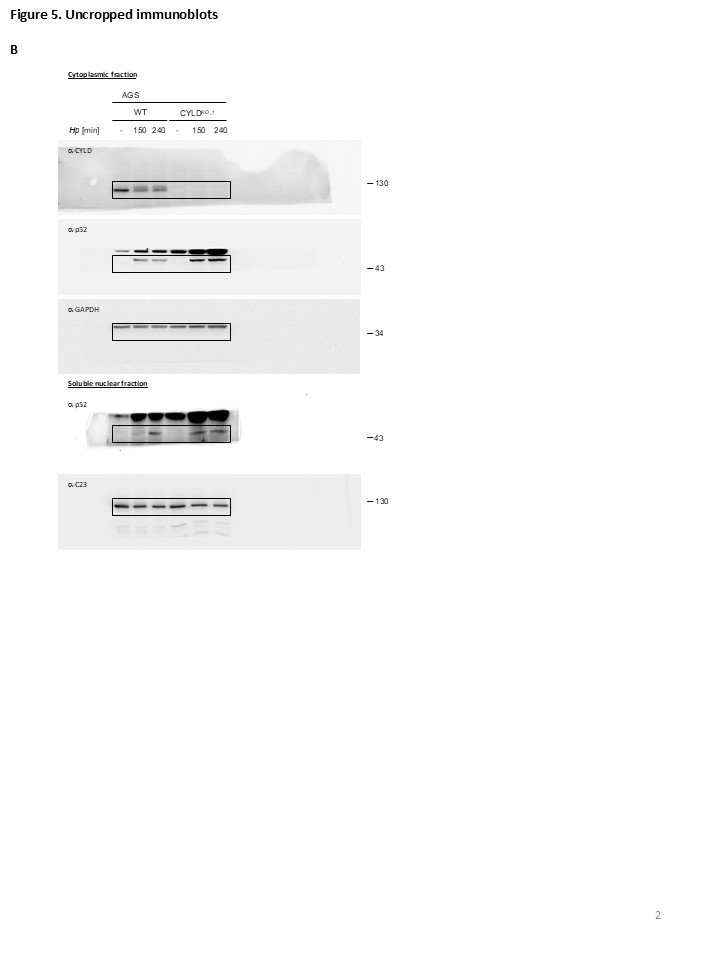

Supplement: Supplementary file 6 — Source data Fig. 5 [file 44319_2025_480_MOESM6_ESM.zip › Source data_Figure 5/Fig 5B.JPG]

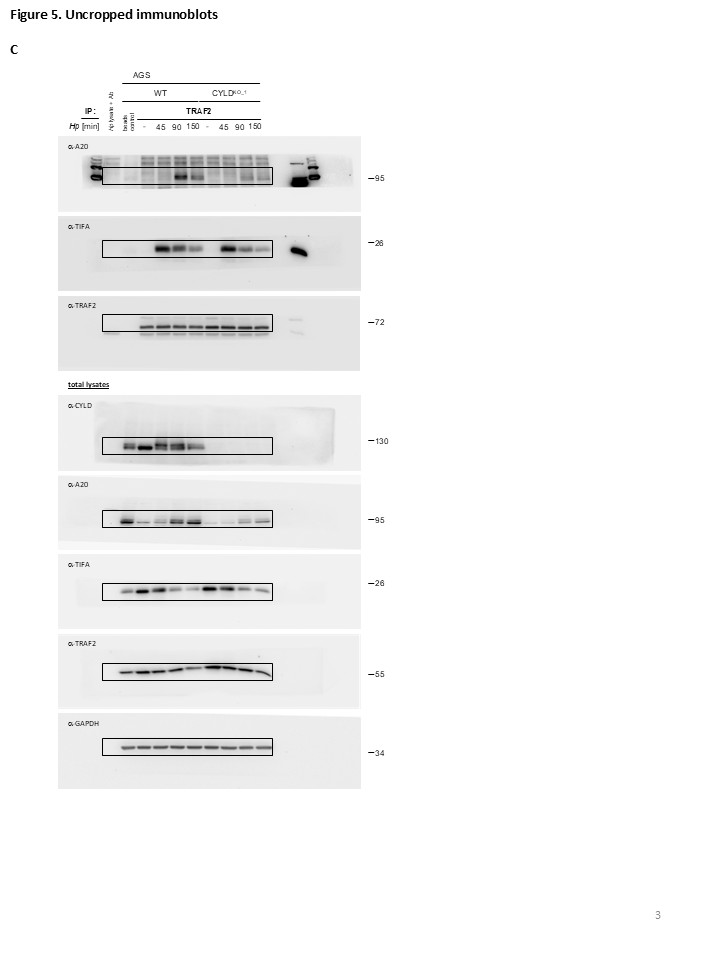

Supplement: Supplementary file 6 — Source data Fig. 5 [file 44319_2025_480_MOESM6_ESM.zip › Source data_Figure 5/Fig 5C.JPG]

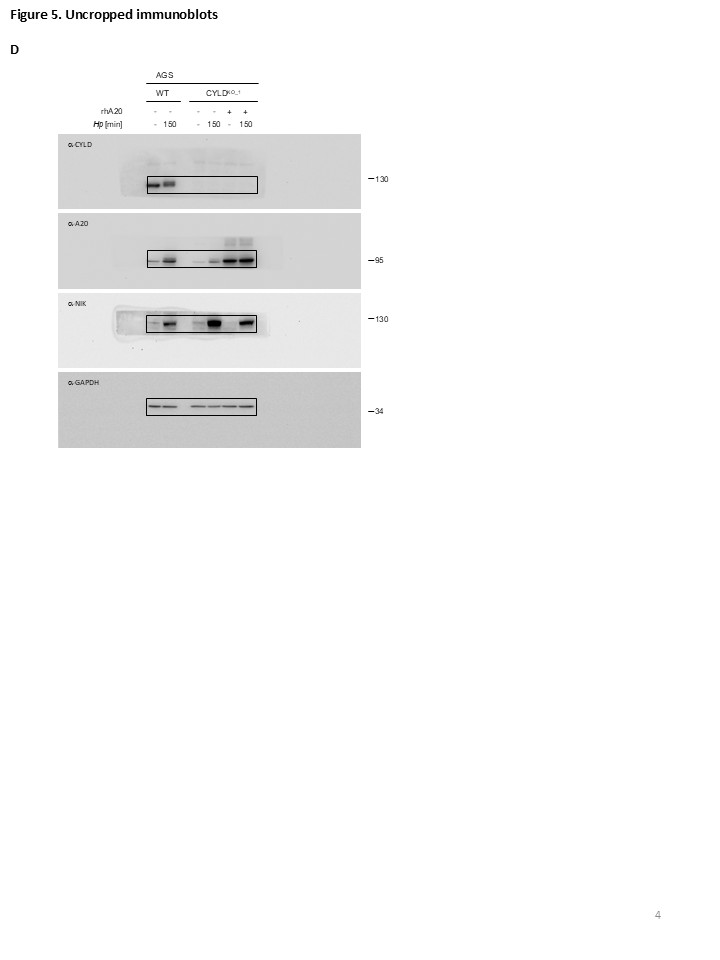

Supplement: Supplementary file 6 — Source data Fig. 5 [file 44319_2025_480_MOESM6_ESM.zip › Source data_Figure 5/Fig 5D.JPG]

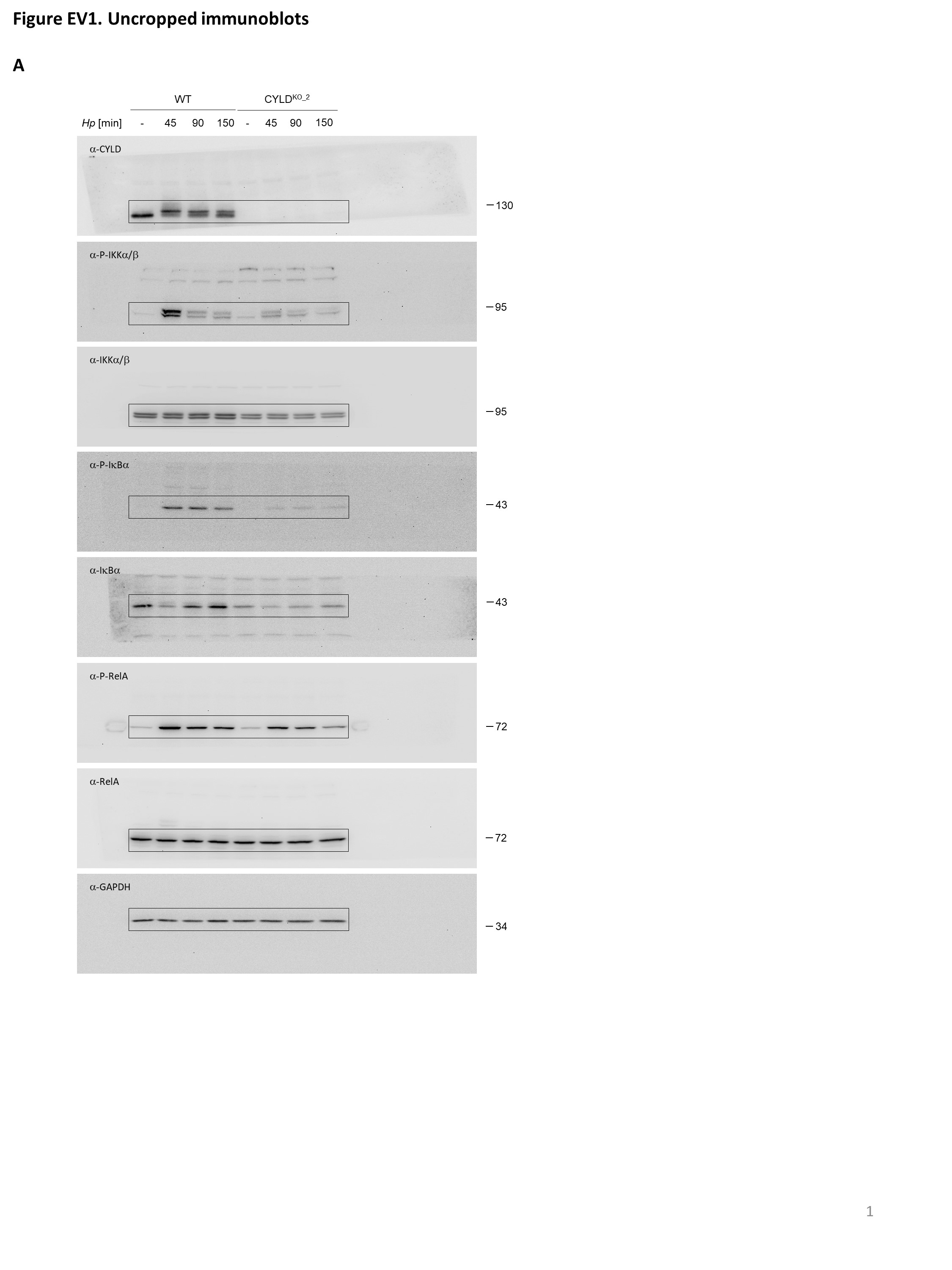

Supplement: Supplementary file 7 — Figure EV1 Source Data [file 44319_2025_480_MOESM7_ESM.zip › Source data_Figure EV1/Fig EV1A.JPG]

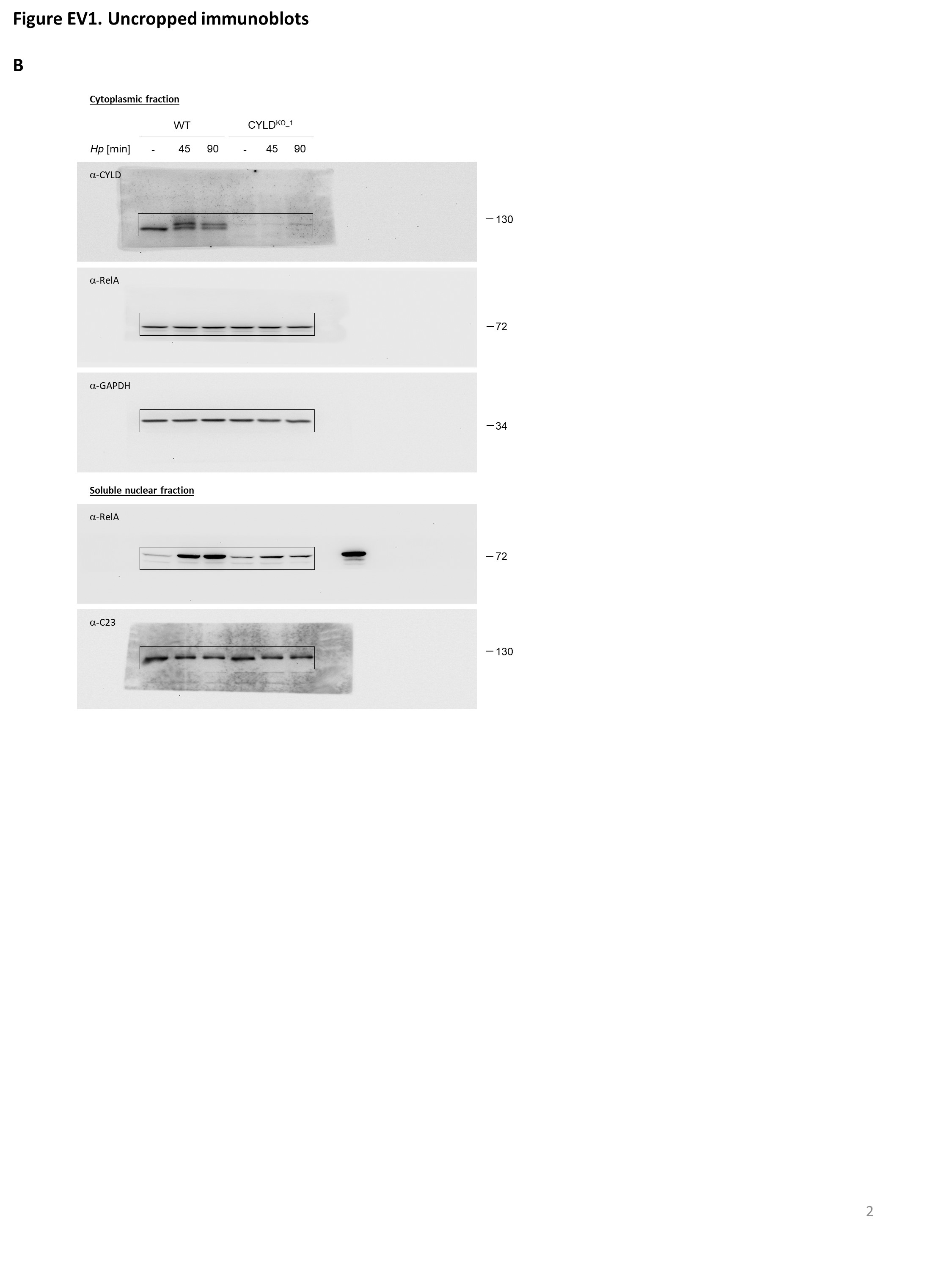

Supplement: Supplementary file 7 — Figure EV1 Source Data [file 44319_2025_480_MOESM7_ESM.zip › Source data_Figure EV1/Fig EV1B.JPG]

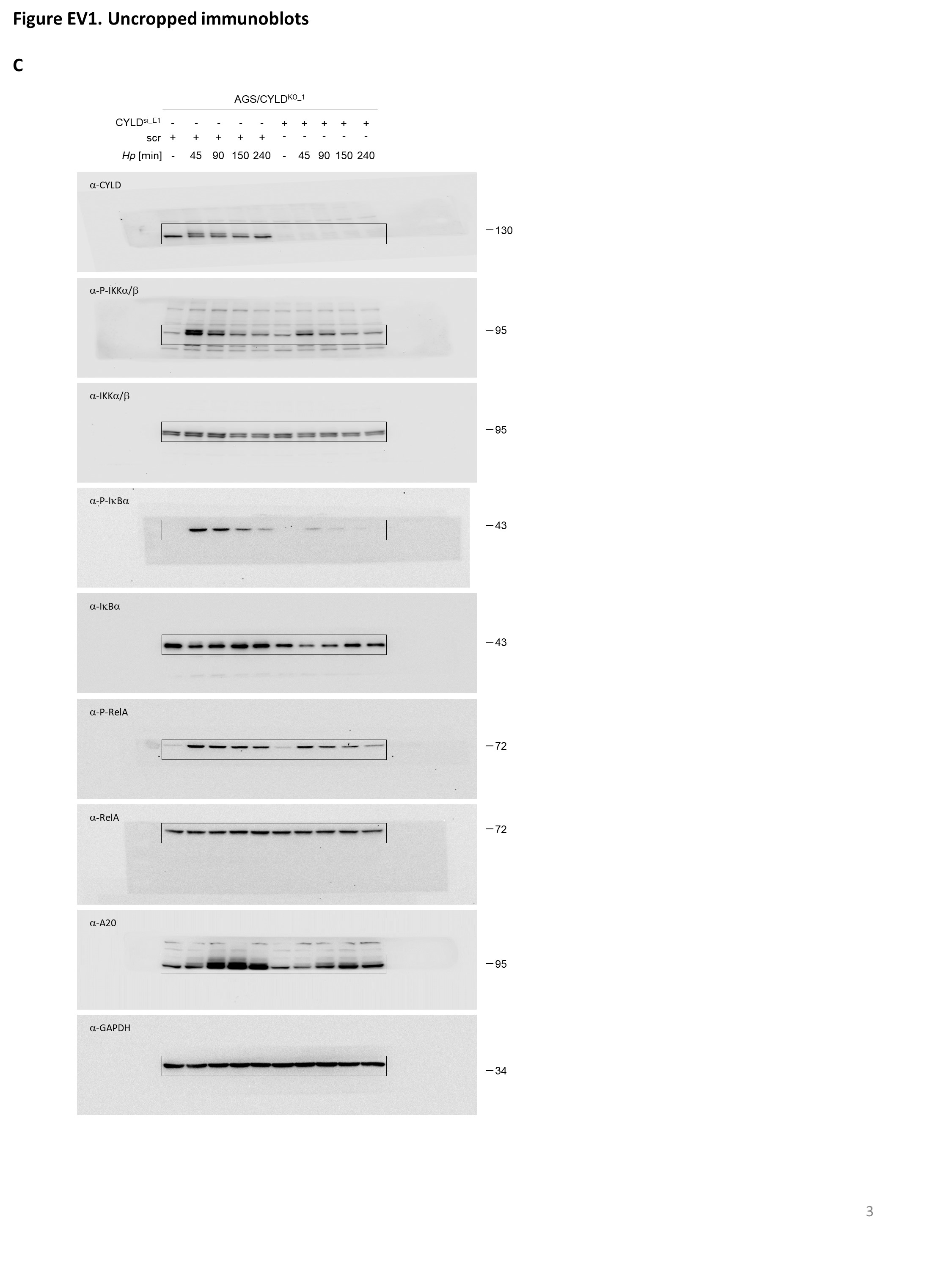

Supplement: Supplementary file 7 — Figure EV1 Source Data [file 44319_2025_480_MOESM7_ESM.zip › Source data_Figure EV1/Fig EV1C.JPG]

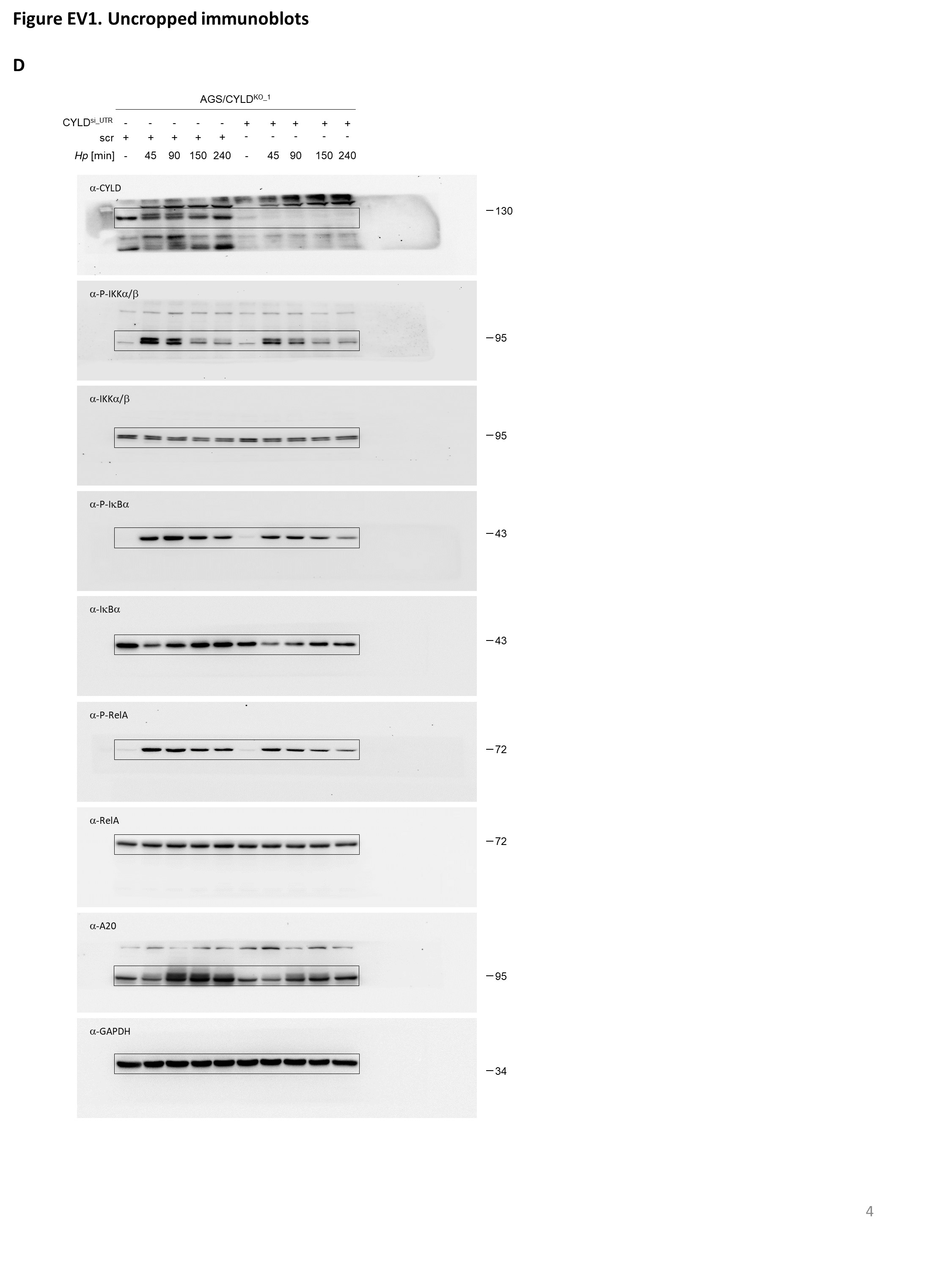

Supplement: Supplementary file 7 — Figure EV1 Source Data [file 44319_2025_480_MOESM7_ESM.zip › Source data_Figure EV1/Fig EV1D.JPG]

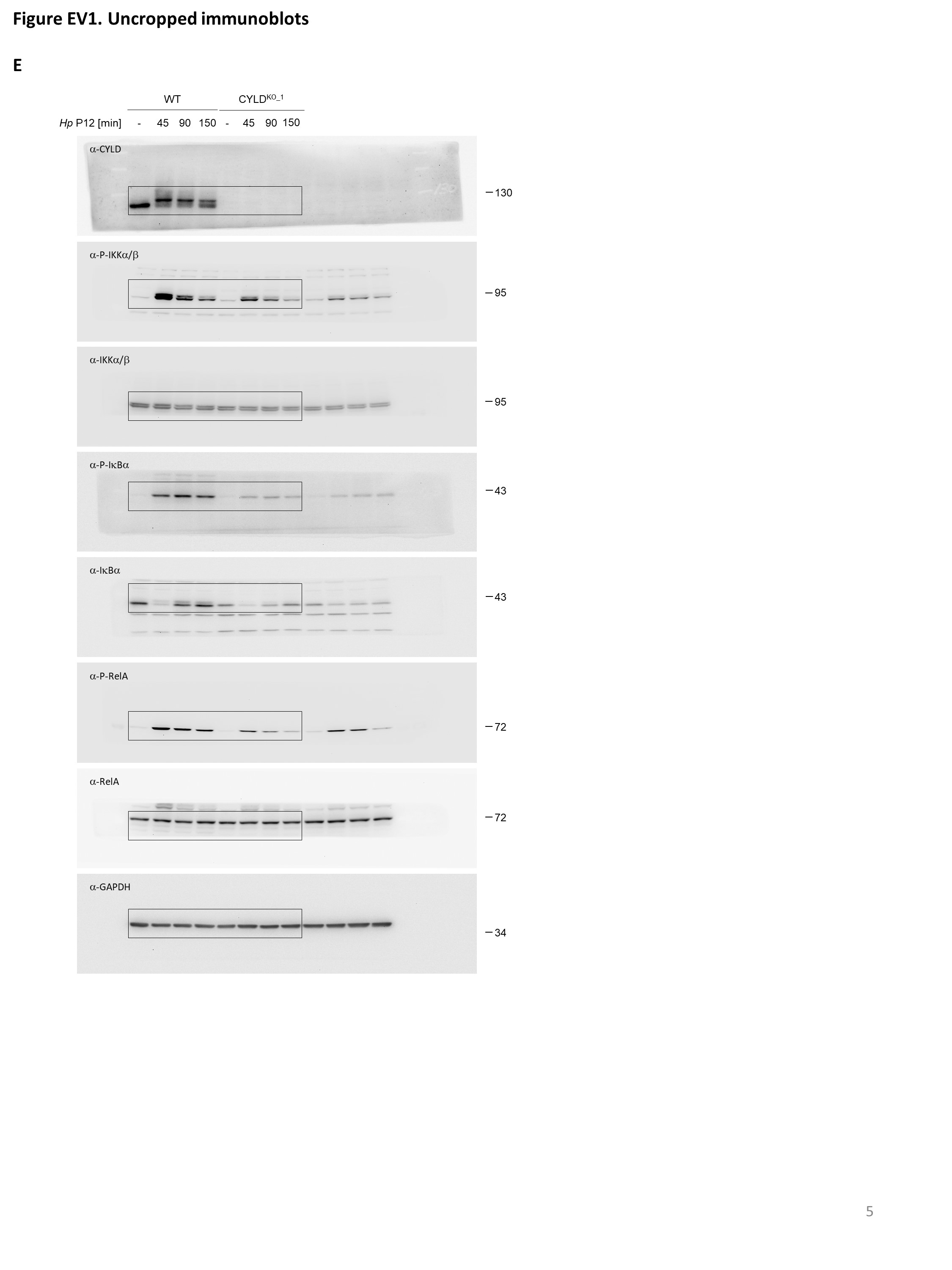

Supplement: Supplementary file 7 — Figure EV1 Source Data [file 44319_2025_480_MOESM7_ESM.zip › Source data_Figure EV1/Fig EV1E.JPG]

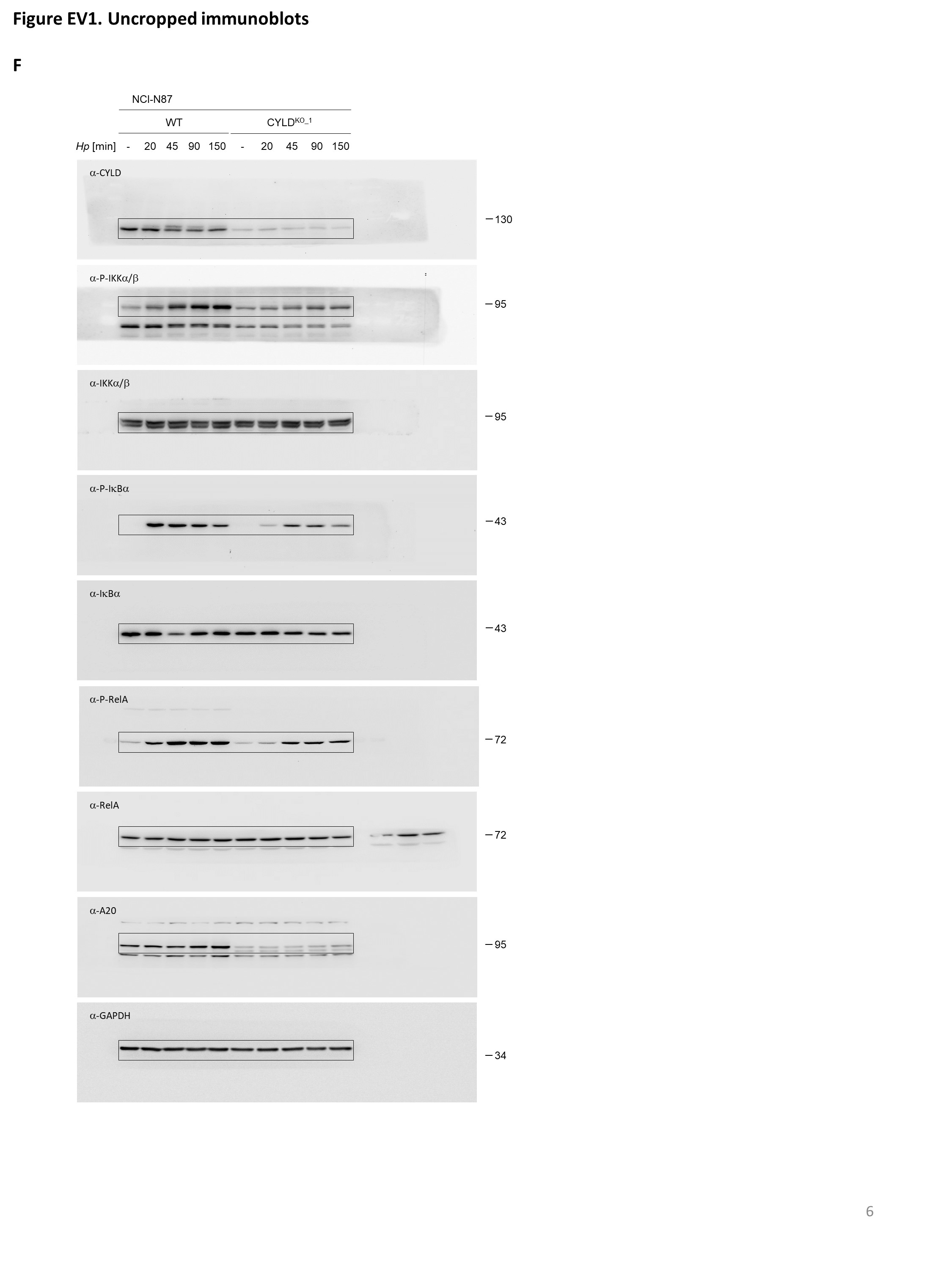

Supplement: Supplementary file 7 — Figure EV1 Source Data [file 44319_2025_480_MOESM7_ESM.zip › Source data_Figure EV1/Fig EV1F.JPG]

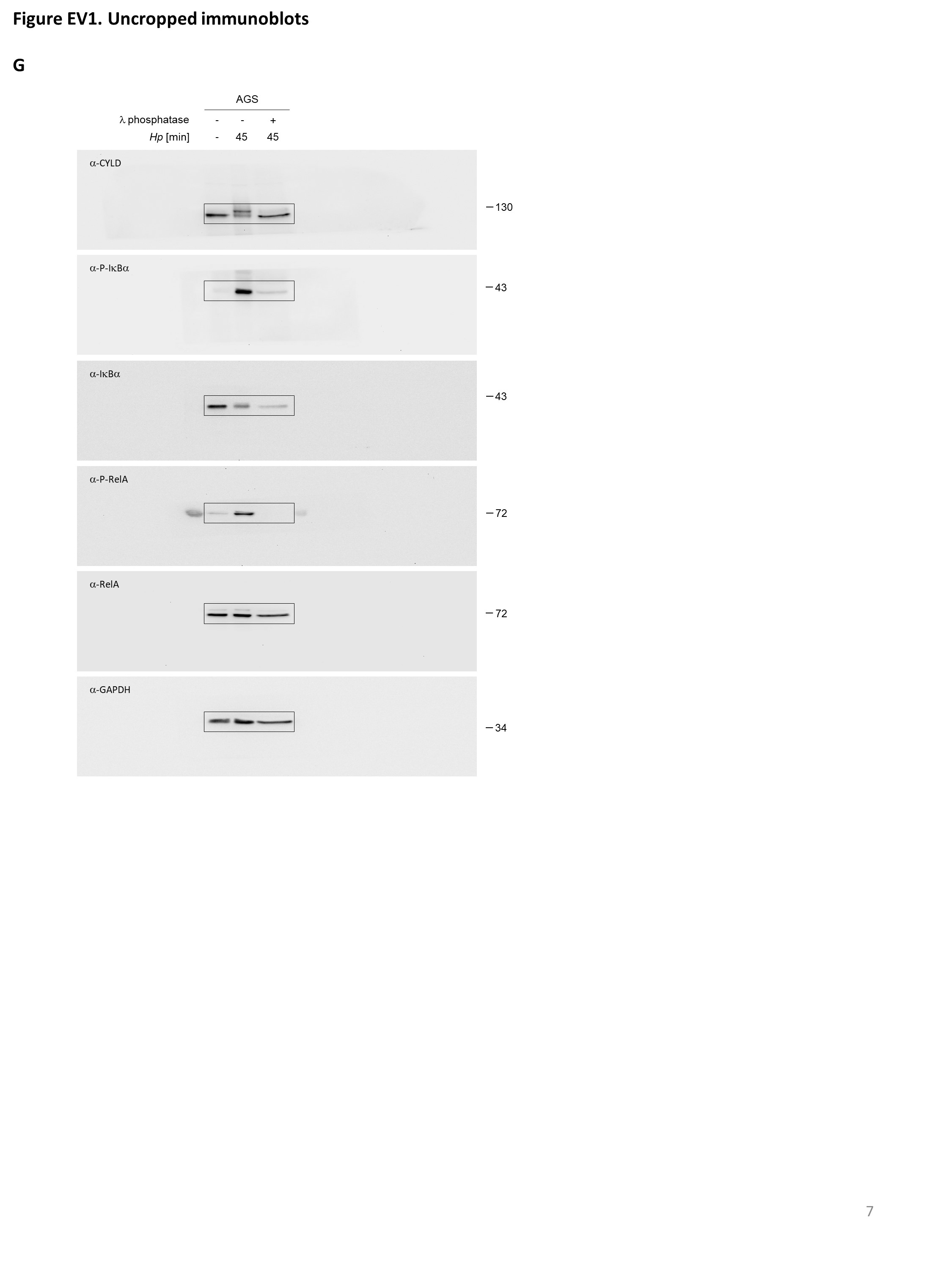

Supplement: Supplementary file 7 — Figure EV1 Source Data [file 44319_2025_480_MOESM7_ESM.zip › Source data_Figure EV1/Fig EV1G.JPG]

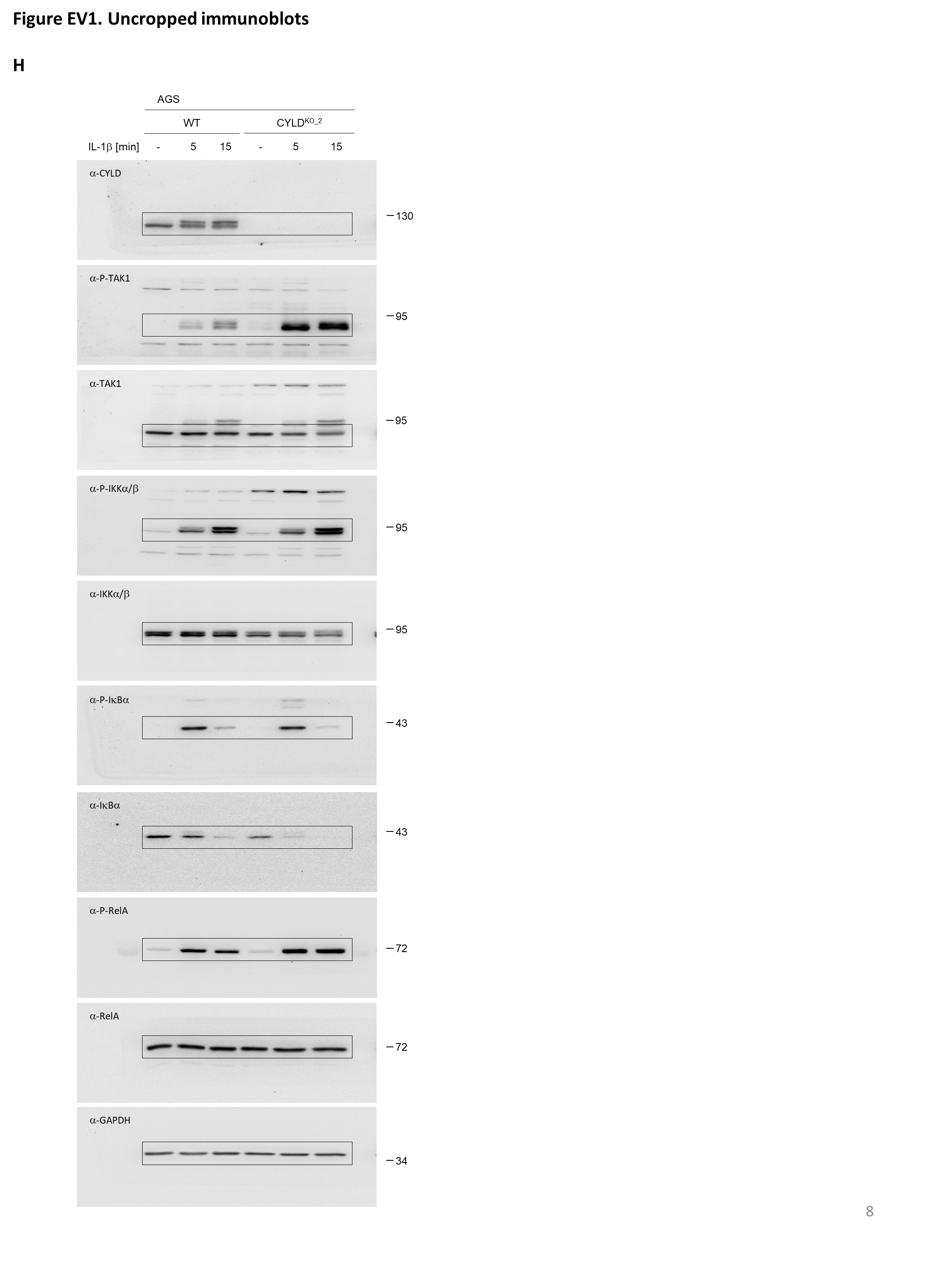

Supplement: Supplementary file 7 — Figure EV1 Source Data [file 44319_2025_480_MOESM7_ESM.zip › Source data_Figure EV1/Fig EV1H.JPG]

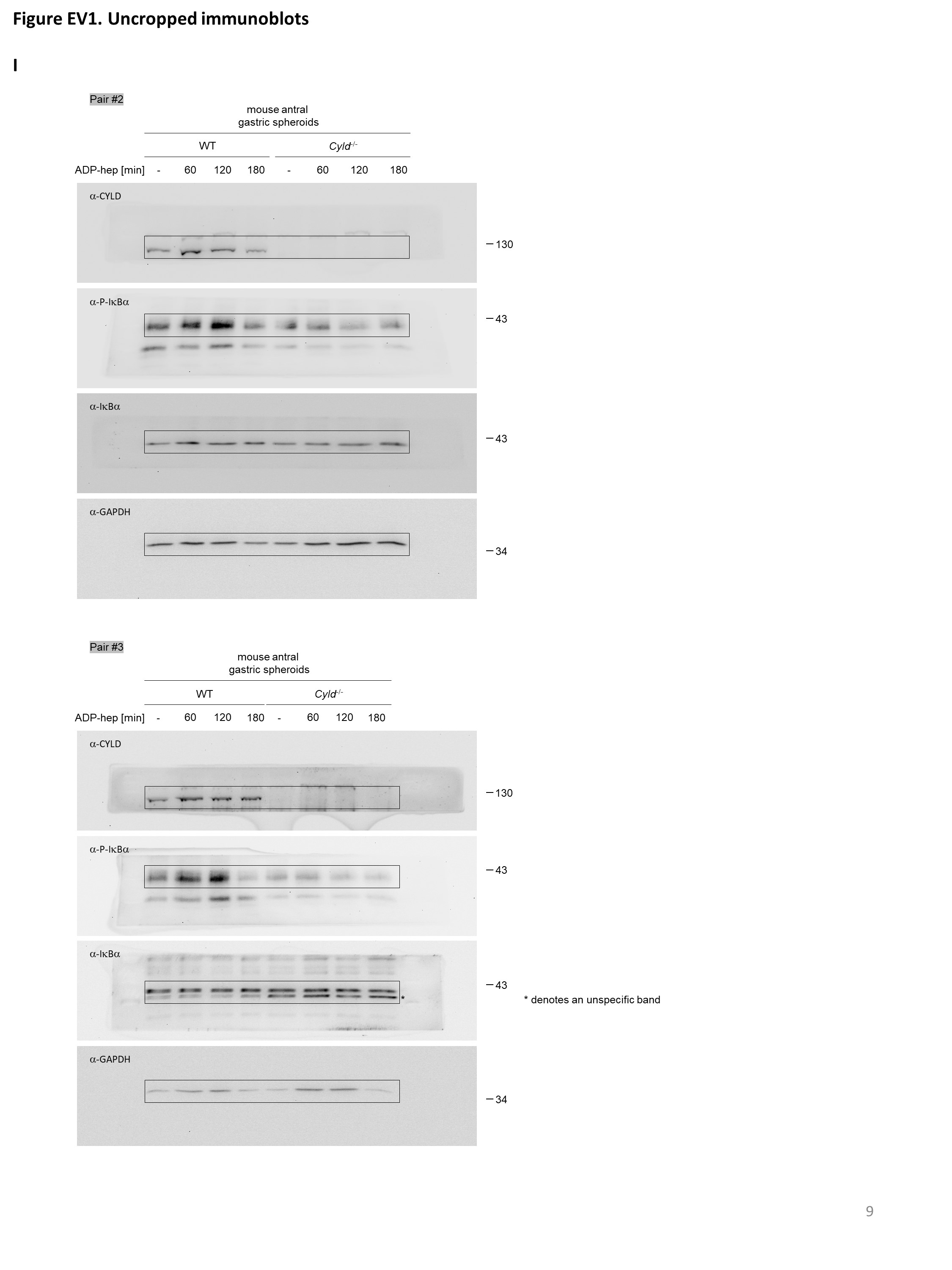

Supplement: Supplementary file 7 — Figure EV1 Source Data [file 44319_2025_480_MOESM7_ESM.zip › Source data_Figure EV1/Fig EV1I.JPG]

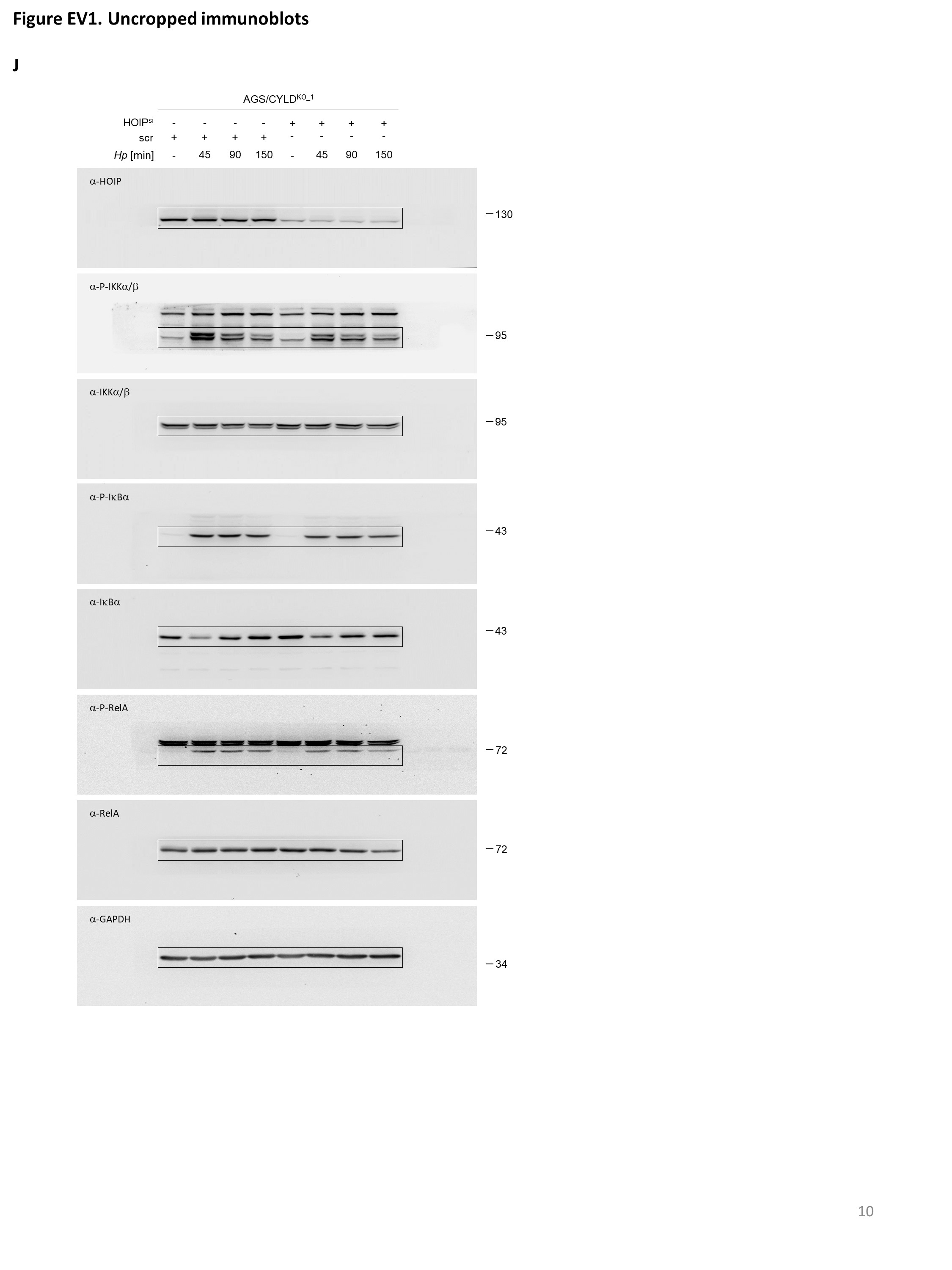

Supplement: Supplementary file 7 — Figure EV1 Source Data [file 44319_2025_480_MOESM7_ESM.zip › Source data_Figure EV1/Fig EV1J.JPG]

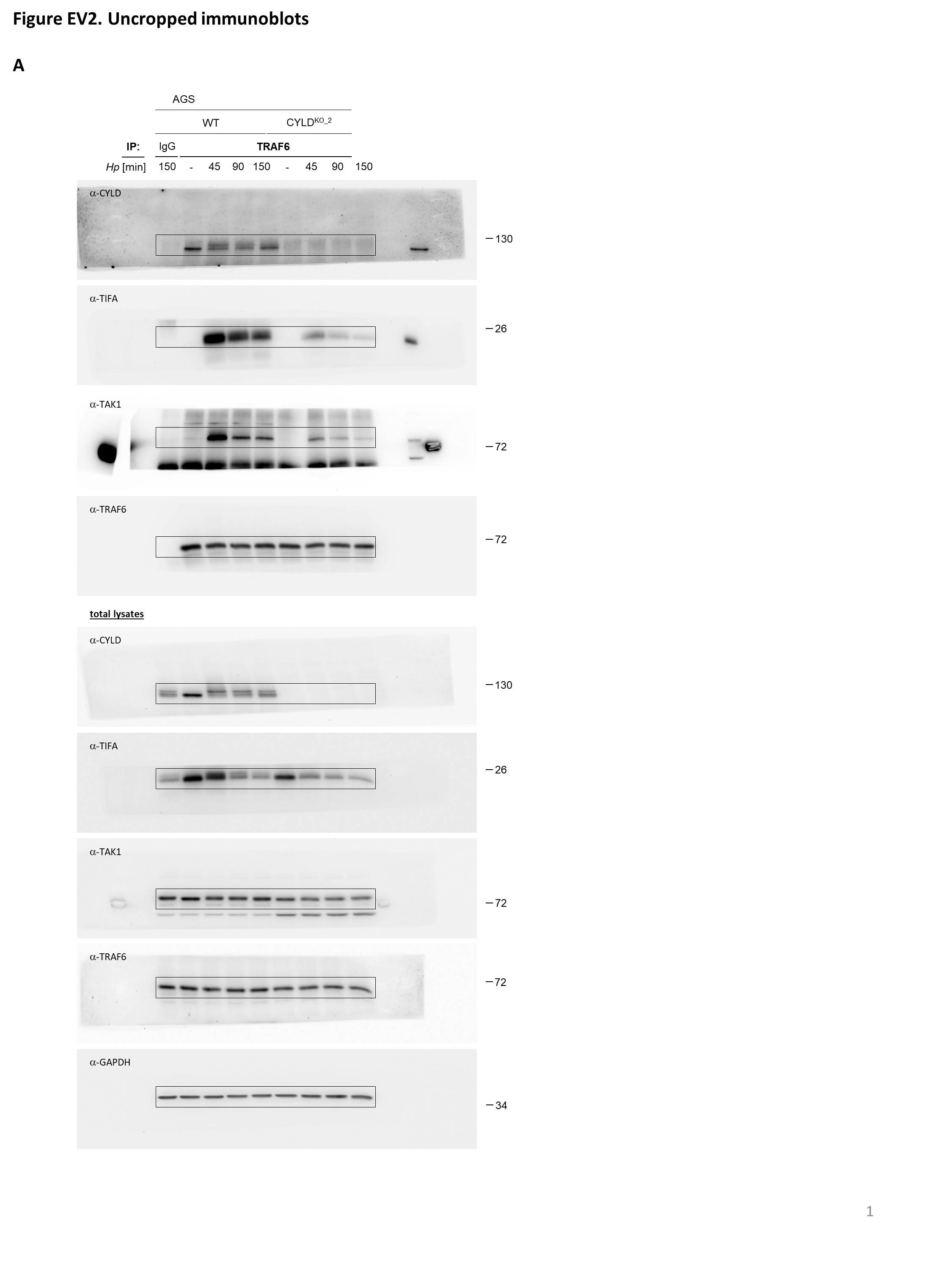

Supplement: Supplementary file 8 — Figure EV2 Source Data [file 44319_2025_480_MOESM8_ESM.zip › Source data_Figure EV2/Fig EV2A.JPG]

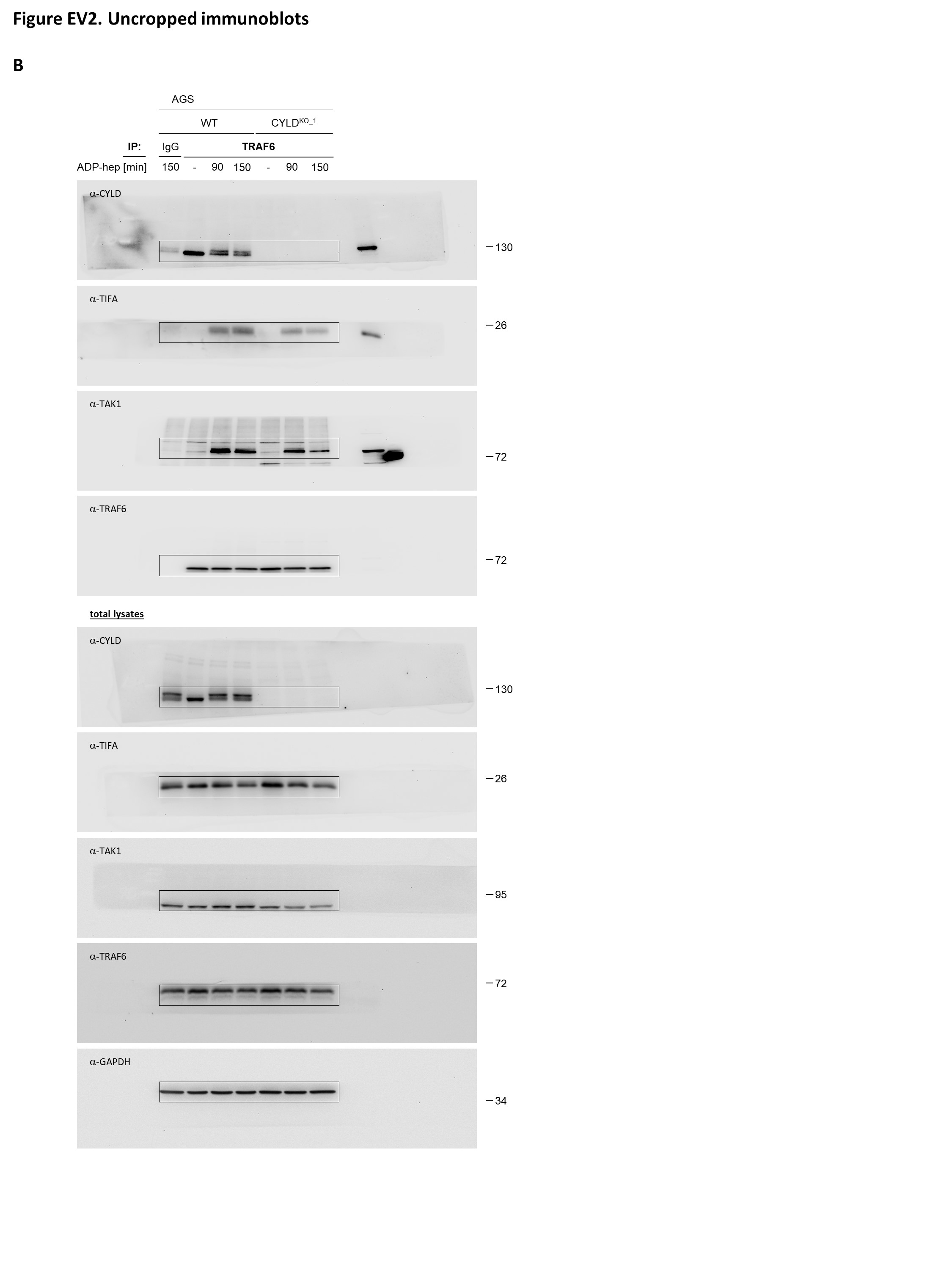

Supplement: Supplementary file 8 — Figure EV2 Source Data [file 44319_2025_480_MOESM8_ESM.zip › Source data_Figure EV2/Fig EV2B.JPG]

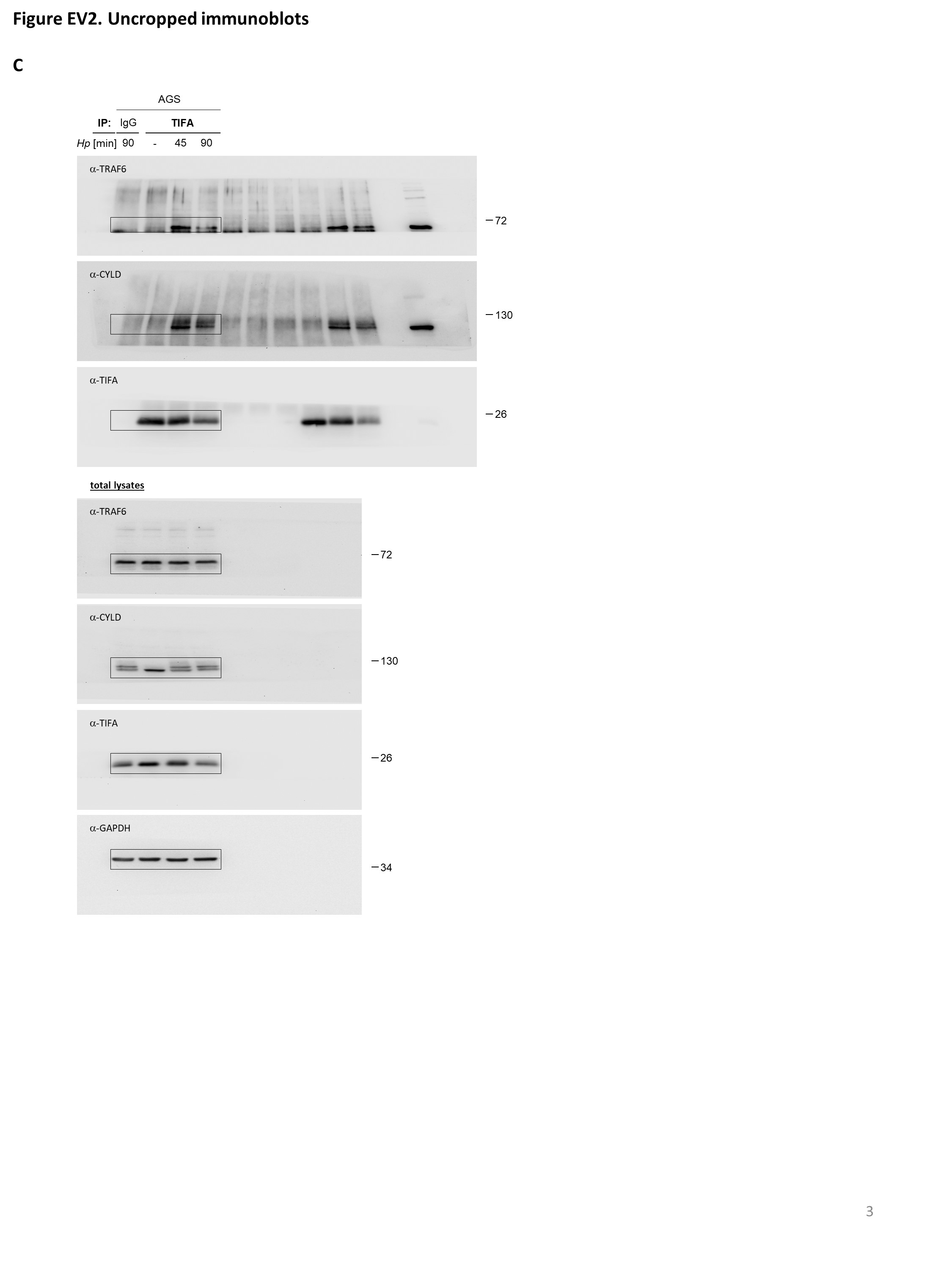

Supplement: Supplementary file 8 — Figure EV2 Source Data [file 44319_2025_480_MOESM8_ESM.zip › Source data_Figure EV2/Fig EV2C.JPG]

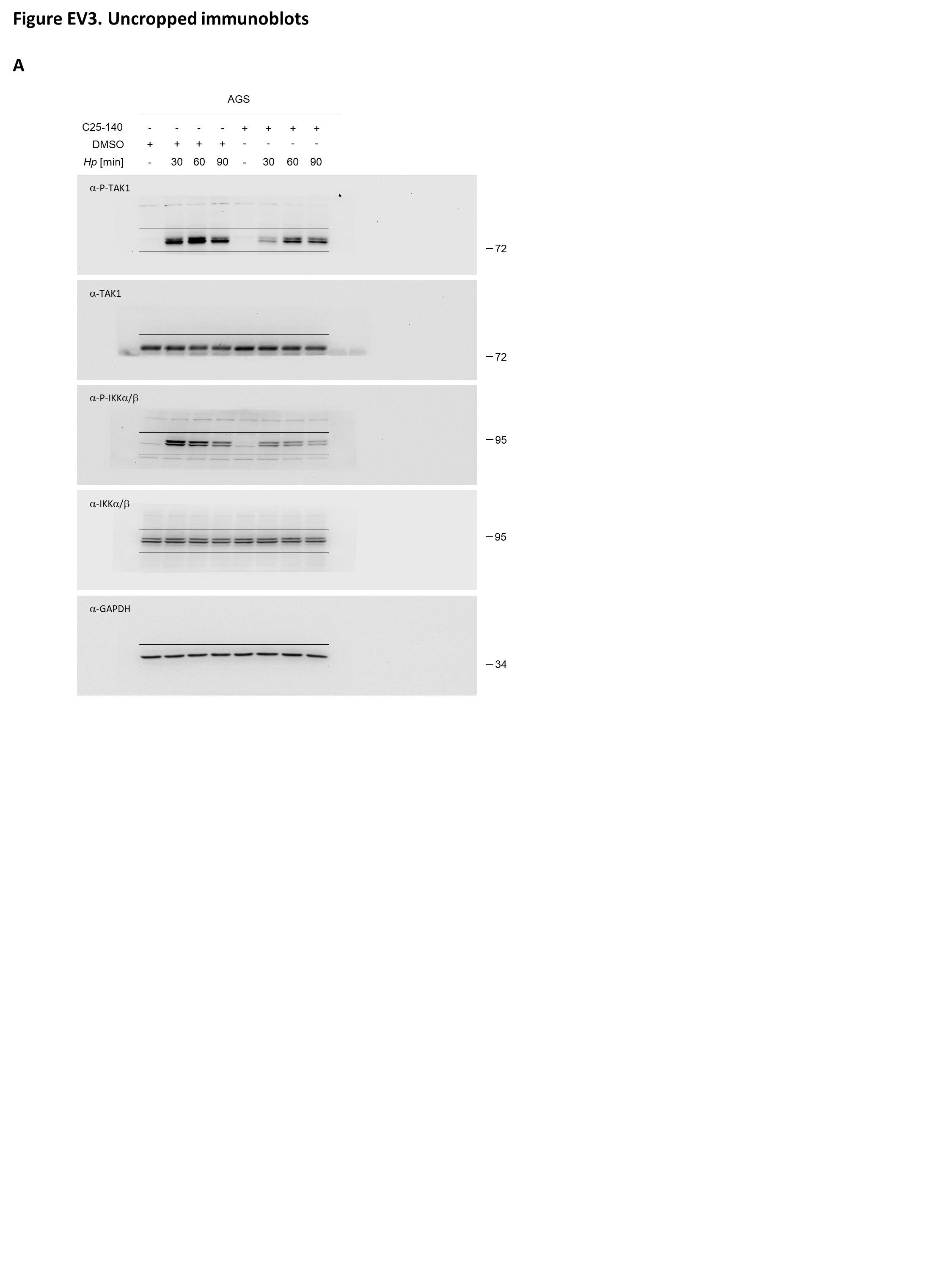

Supplement: Supplementary file 9 — Figure EV3 Source Data [file 44319_2025_480_MOESM9_ESM.zip › Source data_Figure EV3/Fig EV3A.JPG]

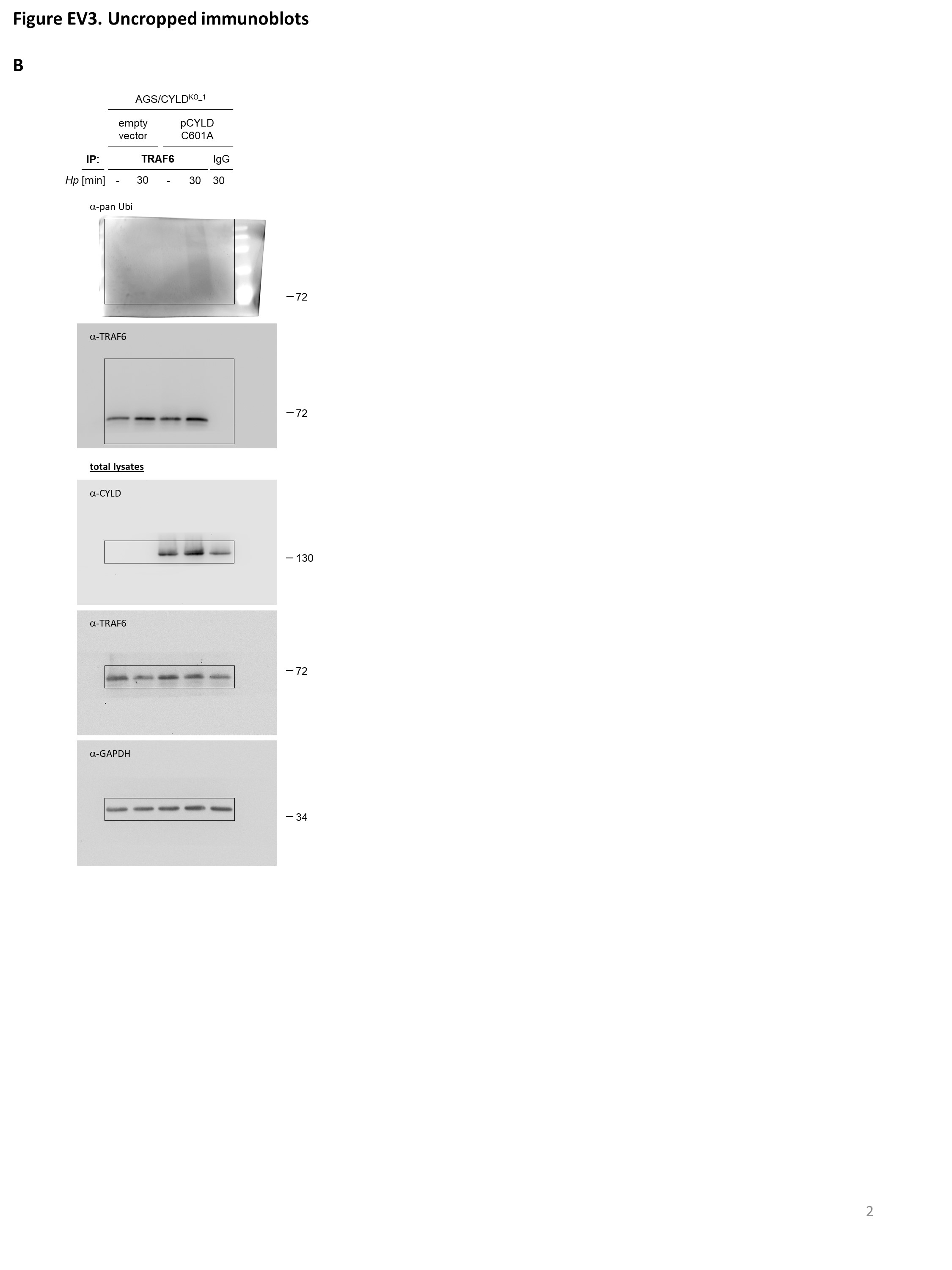

Supplement: Supplementary file 9 — Figure EV3 Source Data [file 44319_2025_480_MOESM9_ESM.zip › Source data_Figure EV3/Fig EV3B.JPG]

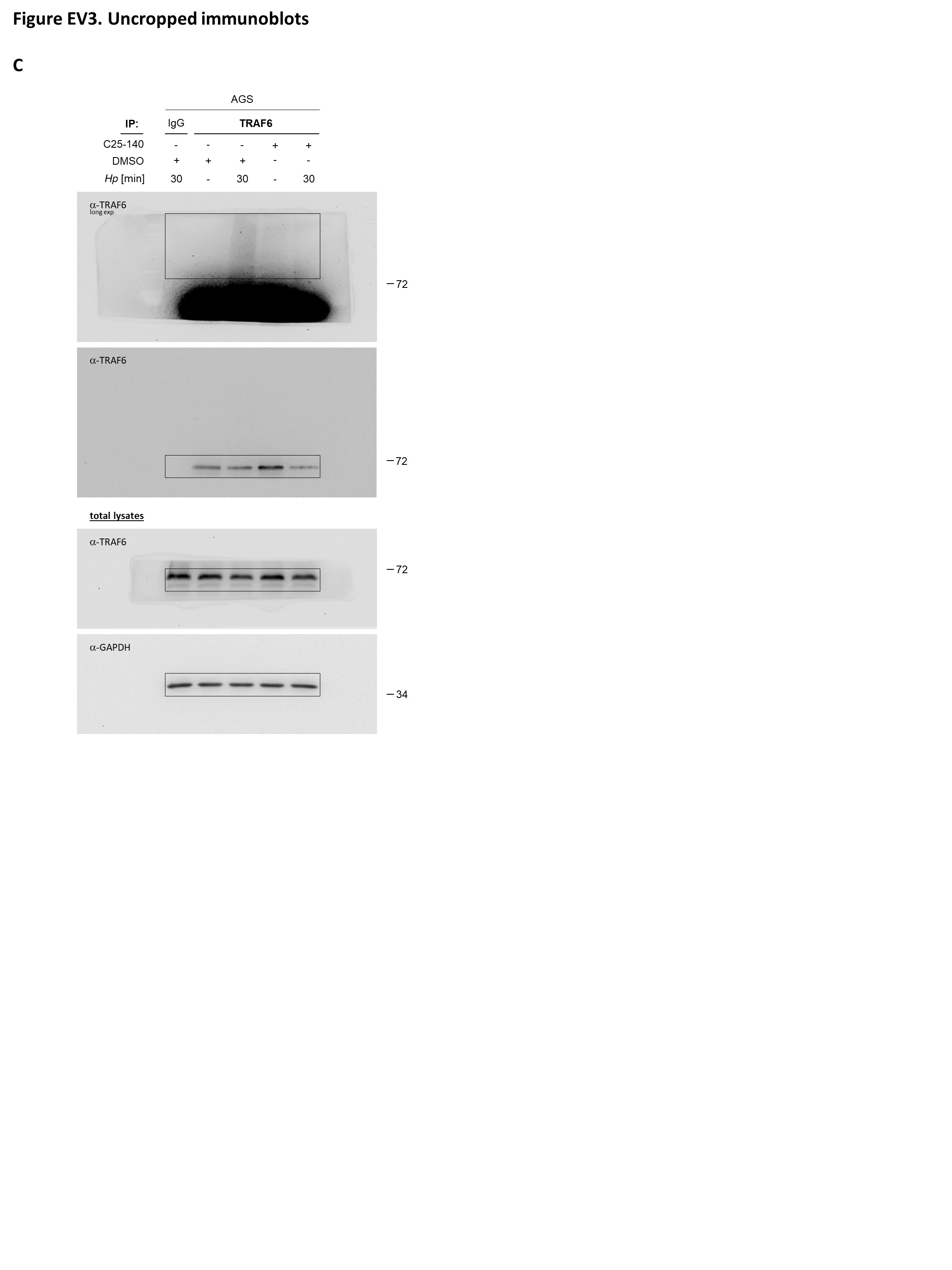

Supplement: Supplementary file 9 — Figure EV3 Source Data [file 44319_2025_480_MOESM9_ESM.zip › Source data_Figure EV3/Fig EV3C.JPG]

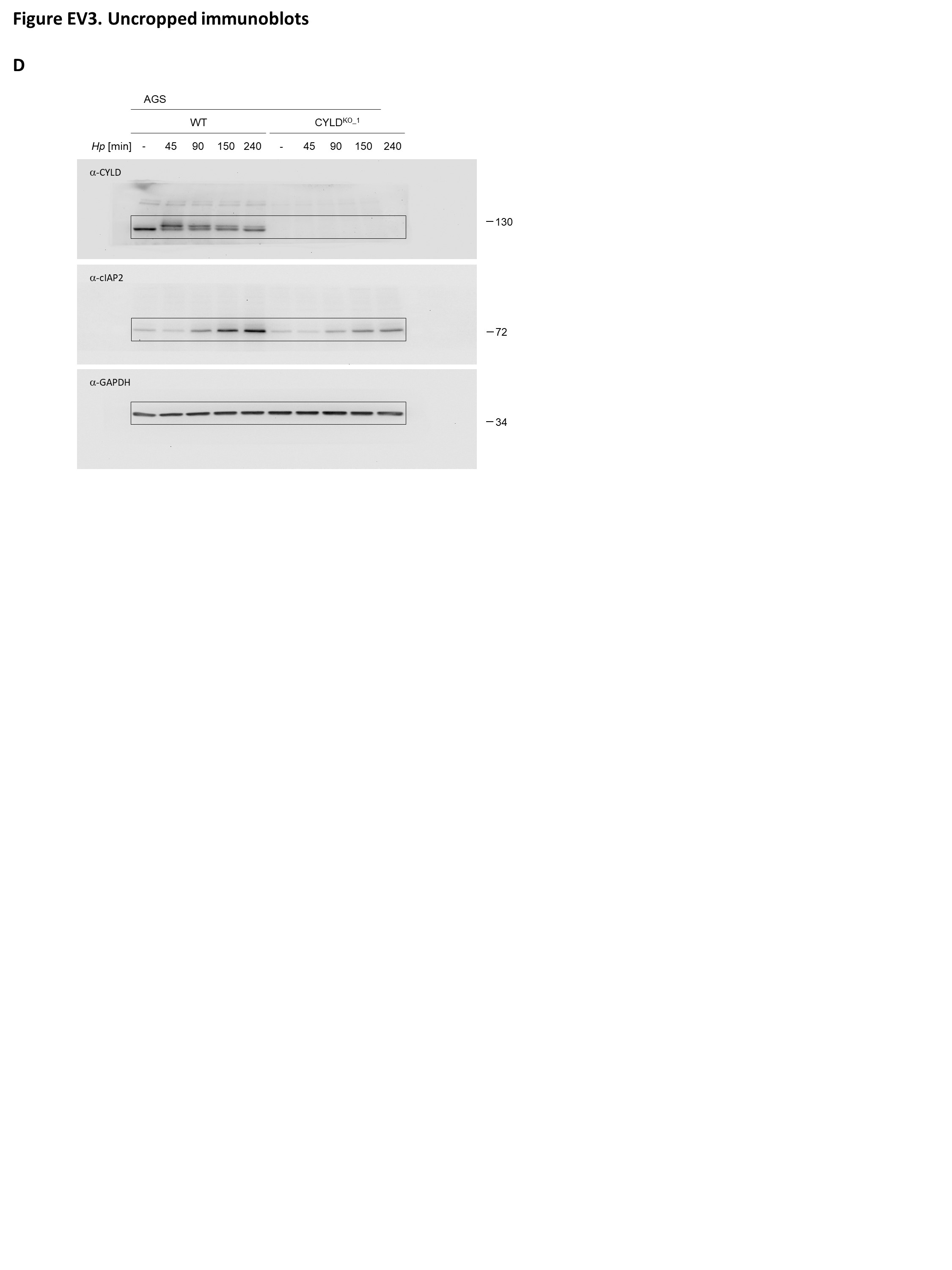

Supplement: Supplementary file 9 — Figure EV3 Source Data [file 44319_2025_480_MOESM9_ESM.zip › Source data_Figure EV3/Fig EV3D.JPG]

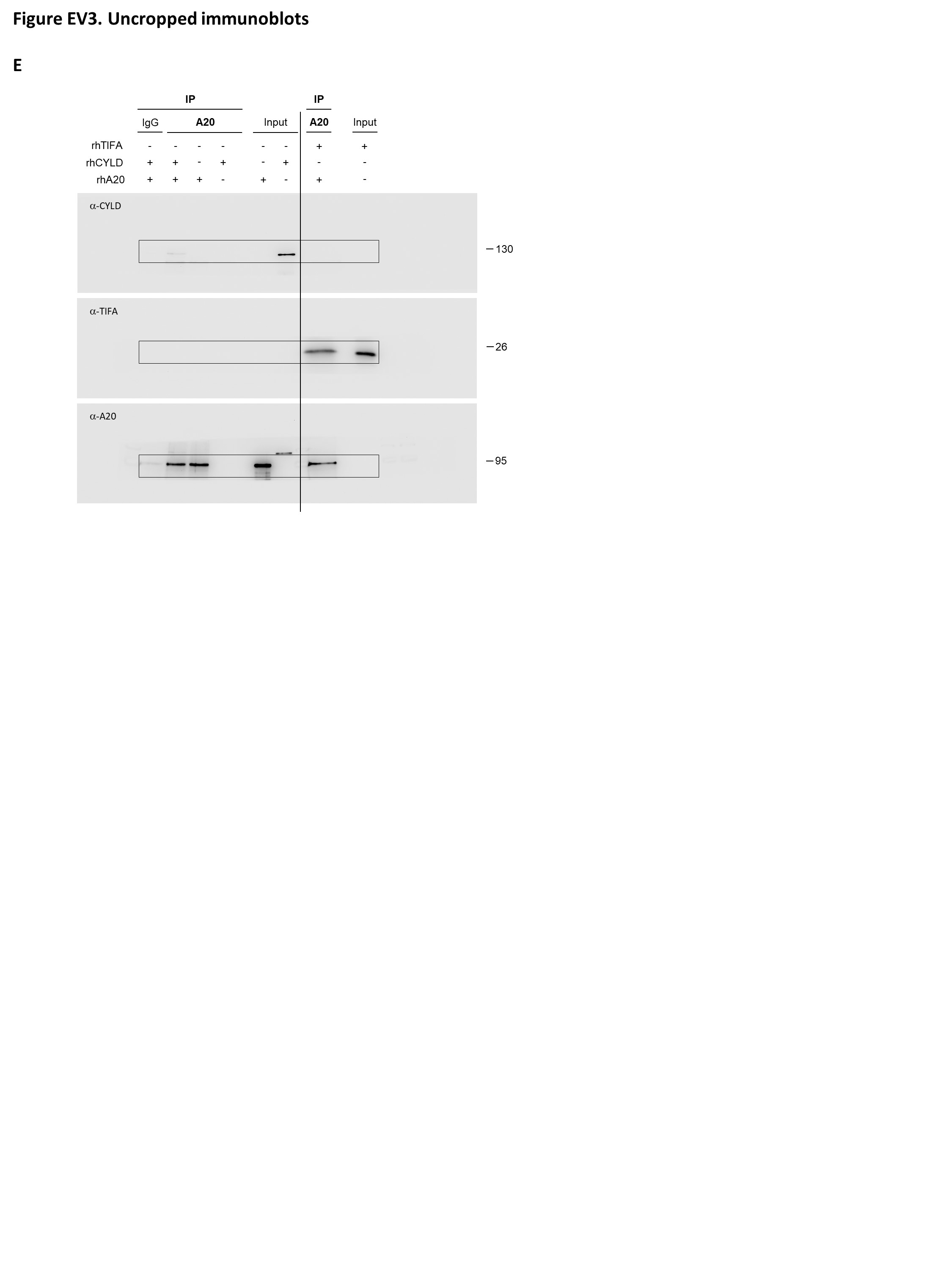

Supplement: Supplementary file 9 — Figure EV3 Source Data [file 44319_2025_480_MOESM9_ESM.zip › Source data_Figure EV3/Fig EV3E.JPG]

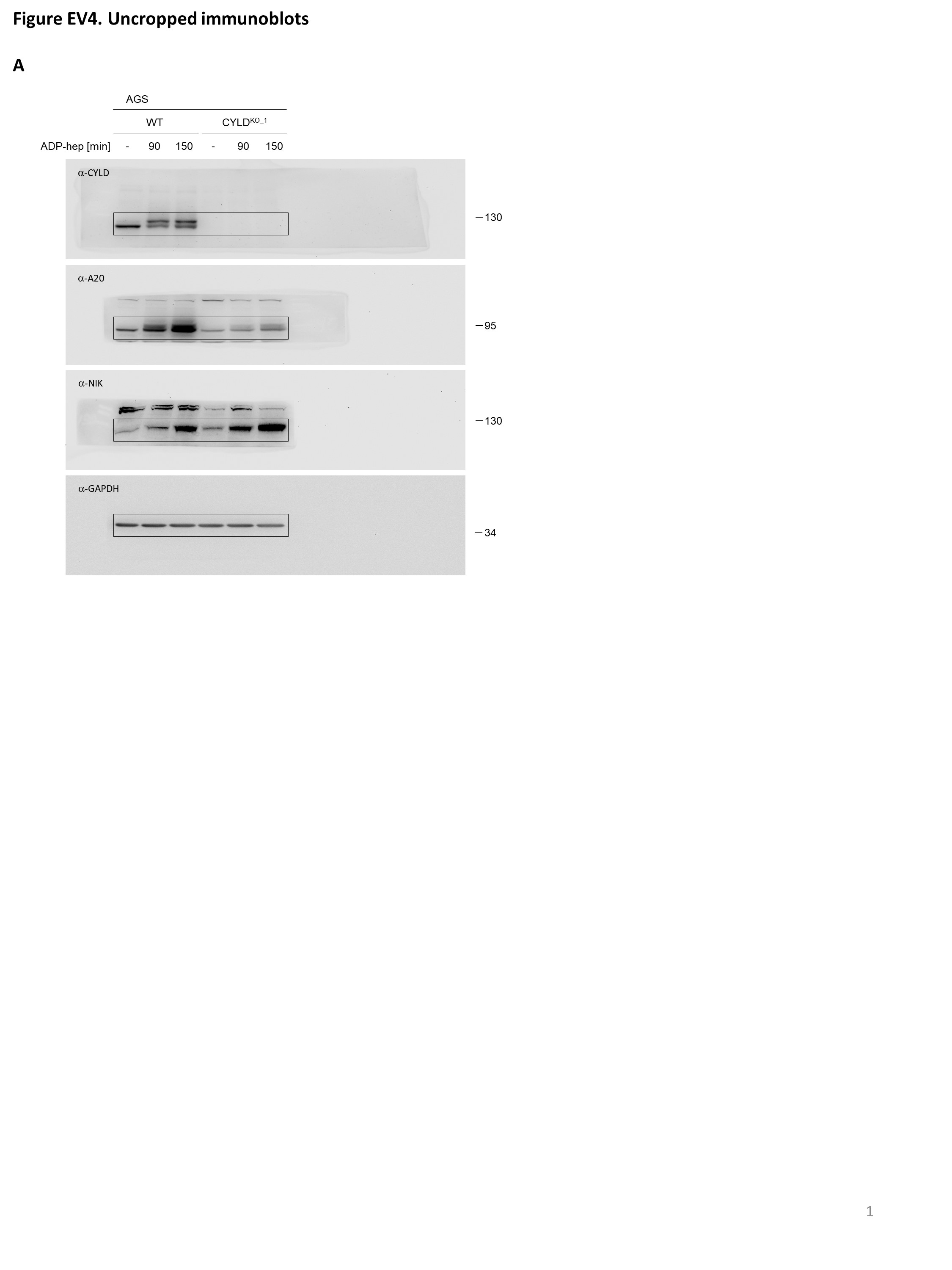

Supplement: Supplementary file 10 — Figure EV4 Source Data [file 44319_2025_480_MOESM10_ESM.zip › Source data_Figure EV4/Fig EV4A.JPG]

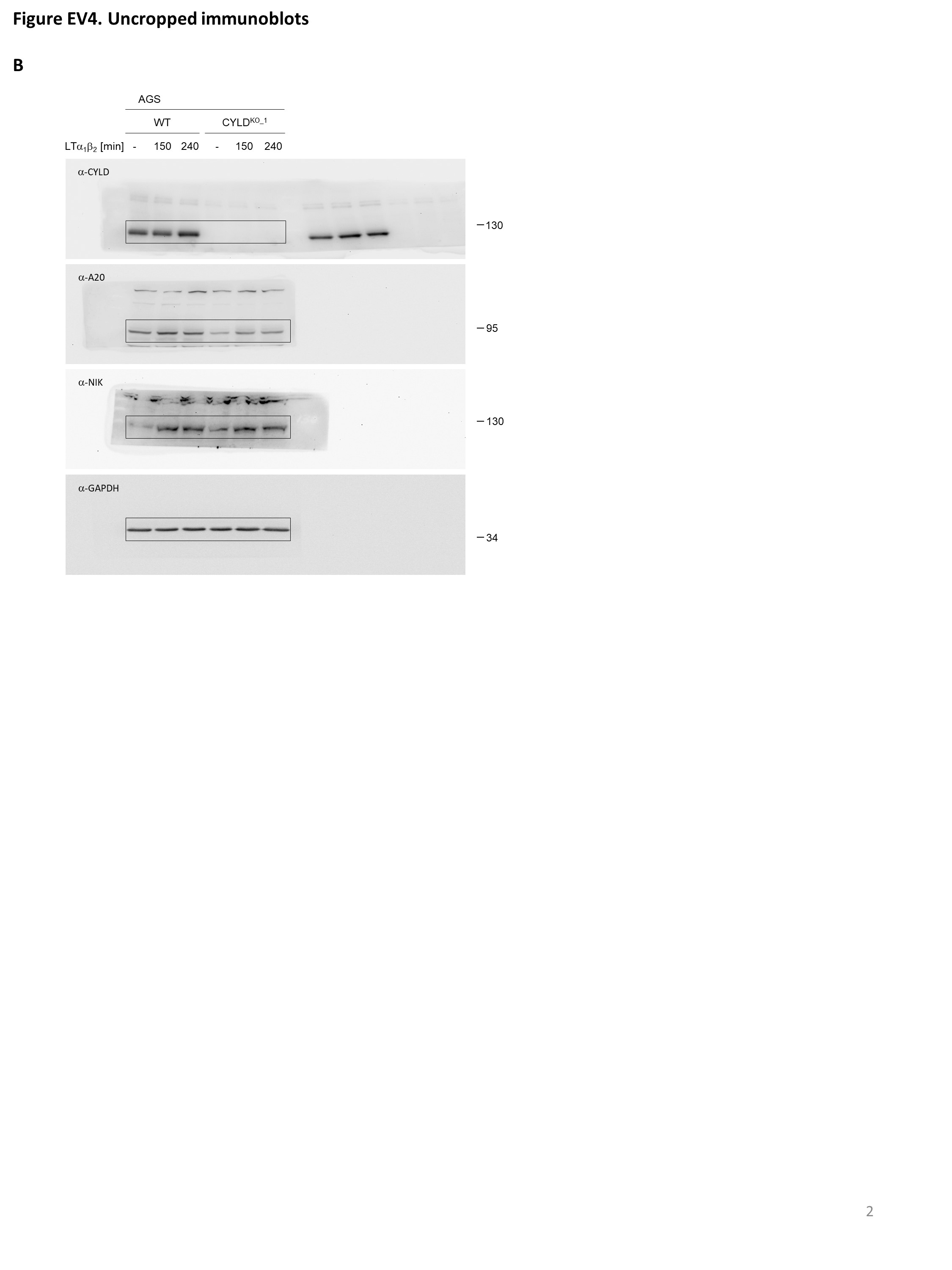

Supplement: Supplementary file 10 — Figure EV4 Source Data [file 44319_2025_480_MOESM10_ESM.zip › Source data_Figure EV4/Fig EV4B.JPG]
