# Supplementary material for: CYLD-TRAF6 interaction promotes ADP-heptose-induced NF-κB signaling in H. pylori infection
Source: EMBO Rep. 2025 May 22;26(13):3241–63. doi: 10.1038/s44319-025-00480-y (PMC12238516; doi:10.1038/s44319-025-00480-y)
Supplement: Supplementary file 11 — Expanded View Figures [file 44319_2025_480_MOESM11_ESM.pdf]

Expanded View Figures

Figure EV1. CYLD supports *H. pylori*-induced classical NF-κB activation.

(**A**) WT and CYLD^{KO_2} AGS cells were left uninfected (-) or infected with *H. pylori* for the times shown. Total lysates were analyzed by immunoblotting using the indicated antibodies. (**B**) WT and CYLD^{KO_2} AGS cells were left uninfected or infected with *H. pylori* for the times shown. Cell fractionation was performed to obtain the cytoplasmic and soluble nuclear fractions before analysis by immunoblotting using the indicated antibodies. GAPDH and C23 serve as the loading controls for the cytoplasmic and soluble nuclear fractions, respectively. (**C**) AGS cells were transfected with siRNAs targeting CYLD (CYLD^{si_E1}, 40 nM) or a non-targeting scrambled siRNA control (scr, 40 nM) 48 h prior to infection with *H. pylori* for the times shown. Total lysates were analyzed by immunoblotting using the indicated antibodies. (**D**) AGS cells were transfected with siRNAs targeting CYLD (CYLD^{si_UTR}, 40 nM) or a non-targeting scrambled siRNA control (scr, 40 nM) 48 h prior to infection with *H. pylori* for the times shown. Total lysates were analyzed by immunoblotting using the indicated antibodies. (**E**) WT and CYLD^{KO_1} AGS cells were left uninfected or infected with *H. pylori* WT P12 strain for the times shown. Total lysates were analyzed by immunoblotting using the indicated antibodies. (**F**) WT and CYLD^{KO_1} NCI-N87 cells were left uninfected or infected with *H. pylori* for the times shown. Total lysates were analyzed by immunoblotting using the indicated antibodies. (**G**) AGS cells were left uninfected or infected with *H. pylori* for 45 min. The *H. pylori*-infected sample was incubated with or without λ phosphatase in the appropriate reaction buffer. Total lysates were analyzed by immunoblotting using the indicated antibodies. (**H**) WT and CYLD^{KO_2} AGS cells were left untreated or treated with 10 ng/ml IL-1β for the indicated times. Total lysates were analyzed by immunoblotting using the indicated antibodies. (**I**) Antral gastric spheroids of two different pairs of mice were left untreated or treated with 500 nM ADP-heptose for the times shown. Total lysates were analyzed by immunoblotting using the indicated antibodies. (**J**) AGS cells were transfected with siRNAs targeting HOIP (HOIP^{si}, 30 nM) or a non-targeting scrambled siRNA control (scr, 30 nM) 48 h prior to infection with *H. pylori* for the times shown. Total lysates were analyzed by immunoblotting using the indicated antibodies. For (**A**, **C–J**), GAPDH serves as the loading control for the total lysates. For all panels, IBs were processed in parallel and depict 1 representative of at least two independent experiments. Asterisk denotes an unspecific band. Source data are available online for this figure.

© The Author(s)

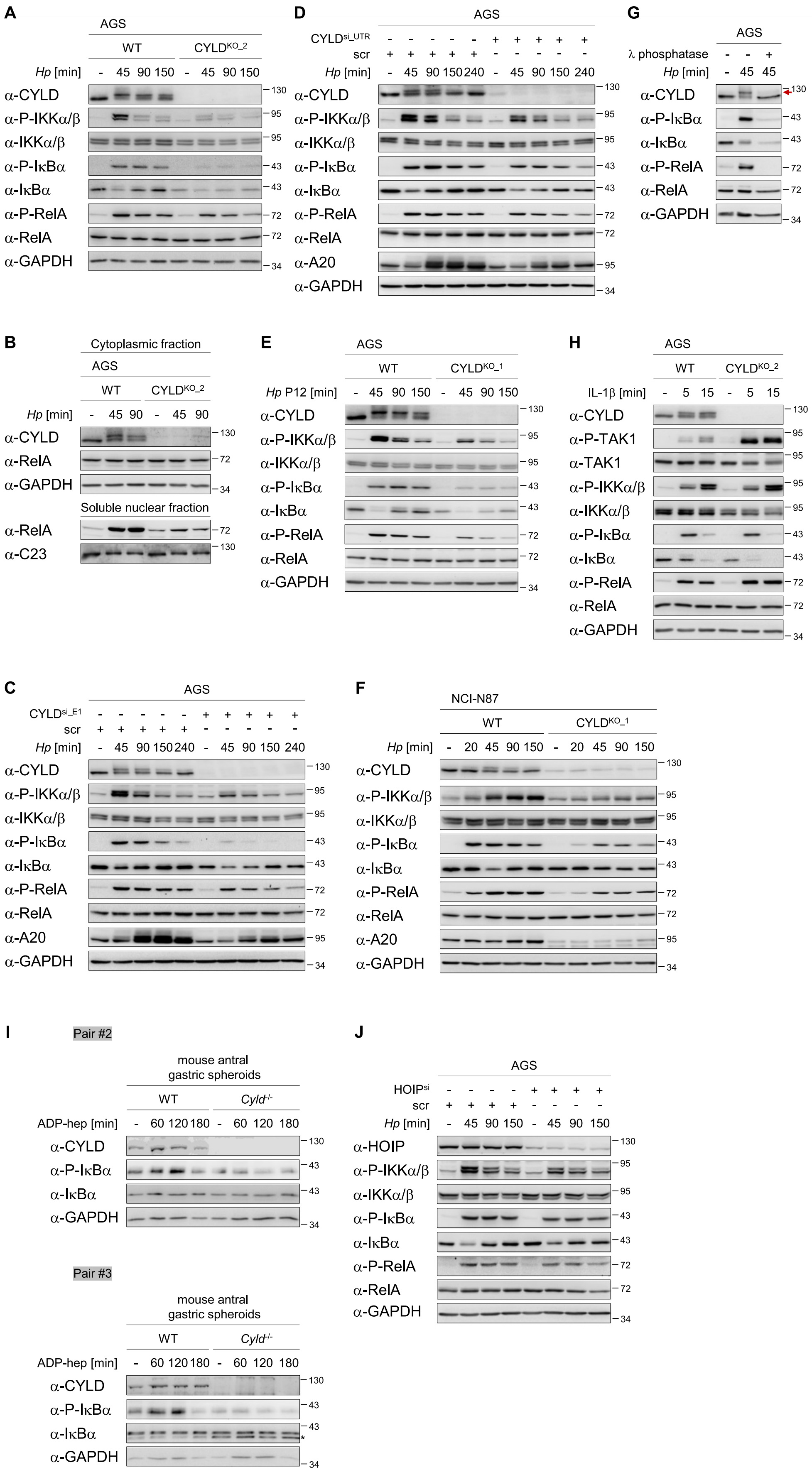
A
AGS
WT
CYLDKO_2
Hp [min] - 45 90 150 - 45 90 150
α-CYLD
α-P-IKKα/β
α-IKKα/β
α-P-IκBα
α-IκBα
α-P-RelA
α-RelA
α-GAPDH
130
95
95
43
43
72
72
34
B
Cytoplasmic fraction
AGS
WT
CYLDKO_2
Hp [min] - 45 90 - 45 90
α-CYLD
α-RelA
α-GAPDH
Soluble nuclear fraction
α-RelA
α-C23
130
72
34
72
130
C
AGS
CYLDsi_E1 - - - - - + + + + +
scr + + + + + - - - - -
Hp [min] - 45 90 150 240 - 45 90 150 240
α-CYLD
α-P-IKKα/β
α-IKKα/β
α-P-IκBα
α-IκBα
α-P-RelA
α-RelA
α-A20
α-GAPDH
130
95
95
43
43
72
72
95
34
D
AGS
CYLDsi_UTR - - - - - + + + + +
scr + + + + + - - - - -
Hp [min] - 45 90 150 240 - 45 90 150 240
α-CYLD
α-P-IKKα/β
α-IKKα/β
α-P-IκBα
α-IκBα
α-P-RelA
α-RelA
α-A20
α-GAPDH
130
95
95
43
43
72
72
95
34
E
AGS
WT
CYLDKO_1
Hp P12 [min] - 45 90 150 - 45 90 150
α-CYLD
α-P-IKKα/β
α-IKKα/β
α-P-IκBα
α-IκBα
α-P-RelA
α-RelA
α-GAPDH
130
95
95
43
43
72
72
34
F
NCI-N87
WT
CYLDKO_1
Hp [min] - 20 45 90 150 - 20 45 90 150
α-CYLD
α-P-IKKα/β
α-IKKα/β
α-P-IκBα
α-IκBα
α-P-RelA
α-RelA
α-A20
α-GAPDH
130
95
95
43
43
72
72
95
34
G
AGS
λ phosphatase - - +
Hp [min] - 45 45
α-CYLD
α-P-IκBα
α-IκBα
α-P-RelA
α-RelA
α-GAPDH
130
43
43
72
72
34
H
AGS
WT
CYLDKO_2
IL-1β [min] - 5 15 - 5 15
α-CYLD
α-P-TAK1
α-TAK1
α-P-IKKα/β
α-IKKα/β
α-P-IκBα
α-IκBα
α-P-RelA
α-RelA
α-GAPDH
130
95
95
95
95
43
43
72
72
34
I
Pair #2
mouse antral gastric spheroids
WT
Cyld-/-
ADP-hep [min] - 60 120 180 - 60 120 180
α-CYLD
α-P-IκBα
α-IκBα
α-GAPDH
130
43
43
34
Pair #3
mouse antral gastric spheroids
WT
Cyld-/-
ADP-hep [min] - 60 120 180 - 60 120 180
α-CYLD
α-P-IκBα
α-IκBα
α-GAPDH
130
43
43
*
34
J
AGS
HOIPsi - - - - + + + +
scr + + + + - - - -
Hp [min] - 45 90 150 - 45 90 150
α-HOIP
α-P-IKKα/β
α-IKKα/β
α-P-IκBα
α-IκBα
α-P-RelA
α-RelA
α-GAPDH
130
95
95
43
43
72
72
34

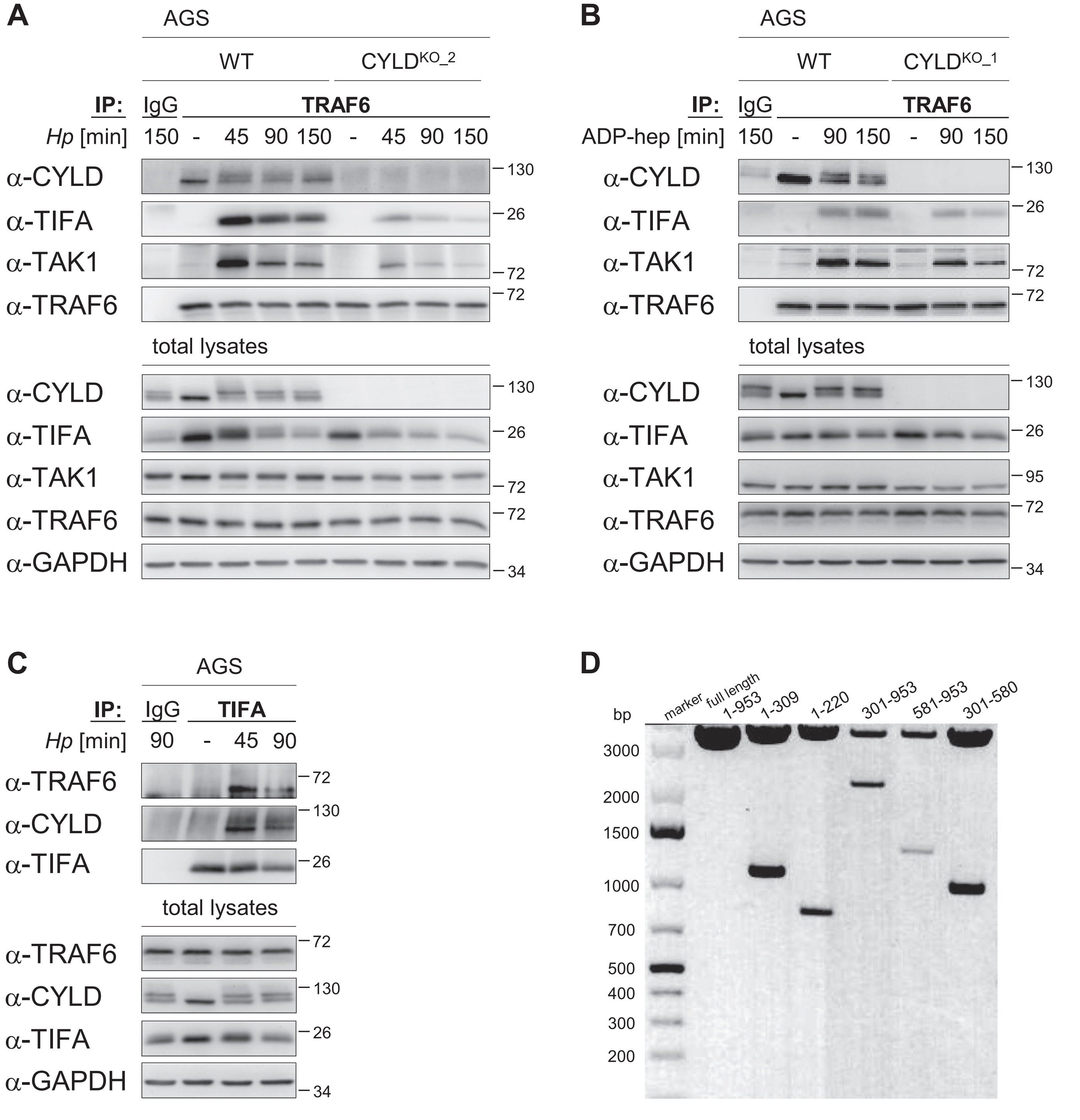

Figure EV2. CYLD associates with the TIFA/TRAF6/TAK1 complex upon *H. pylori* infection.

(**A**) WT and CYLD^{KO_2} AGS cells were left uninfected (−) or infected with *H. pylori* for the times shown and harvested for total lysates. IP with an anti-TRAF6 antibody or isotype-matched antibody (IgG) was performed. Eluates and total lysates were analyzed by immunoblotting using the indicated antibodies. (**B**) WT and CYLD^{KO_1} AGS cells were left untreated or treated with 200 nM ADP-heptose for the times shown and harvested for total lysates. IP with an anti-TRAF6 antibody or isotype-matched antibody (IgG) was performed. Eluates and total lysates were analyzed by immunoblotting using the indicated antibodies. (**C**) AGS cells were left uninfected or infected with *H. pylori* for the times shown and harvested for total lysates. IP with an anti-TIFA antibody or isotype-matched antibody (IgG) was performed. Eluates and total lysates were analyzed by immunoblotting using the indicated antibodies. (**D**) Full-length and the five truncated variants of human CYLD isoform 2 were digested with BamHI/NheI und analyzed on a 1% agarose gel containing GelRed®. The expected fragment sizes for the restriction digests of the full-length and CYLD variants were (in bp): full-length: 3366, 2975; 1–309: 3366, 1037; 1–220: 3366, 779; 301–953: 3366, 2075; 581–953: 3366, 1244; 301–580: 3366, 959. For (**A–C**), IBs were processed in parallel and depict 1 representative of at least two independent experiments. Source data are available online for this figure.

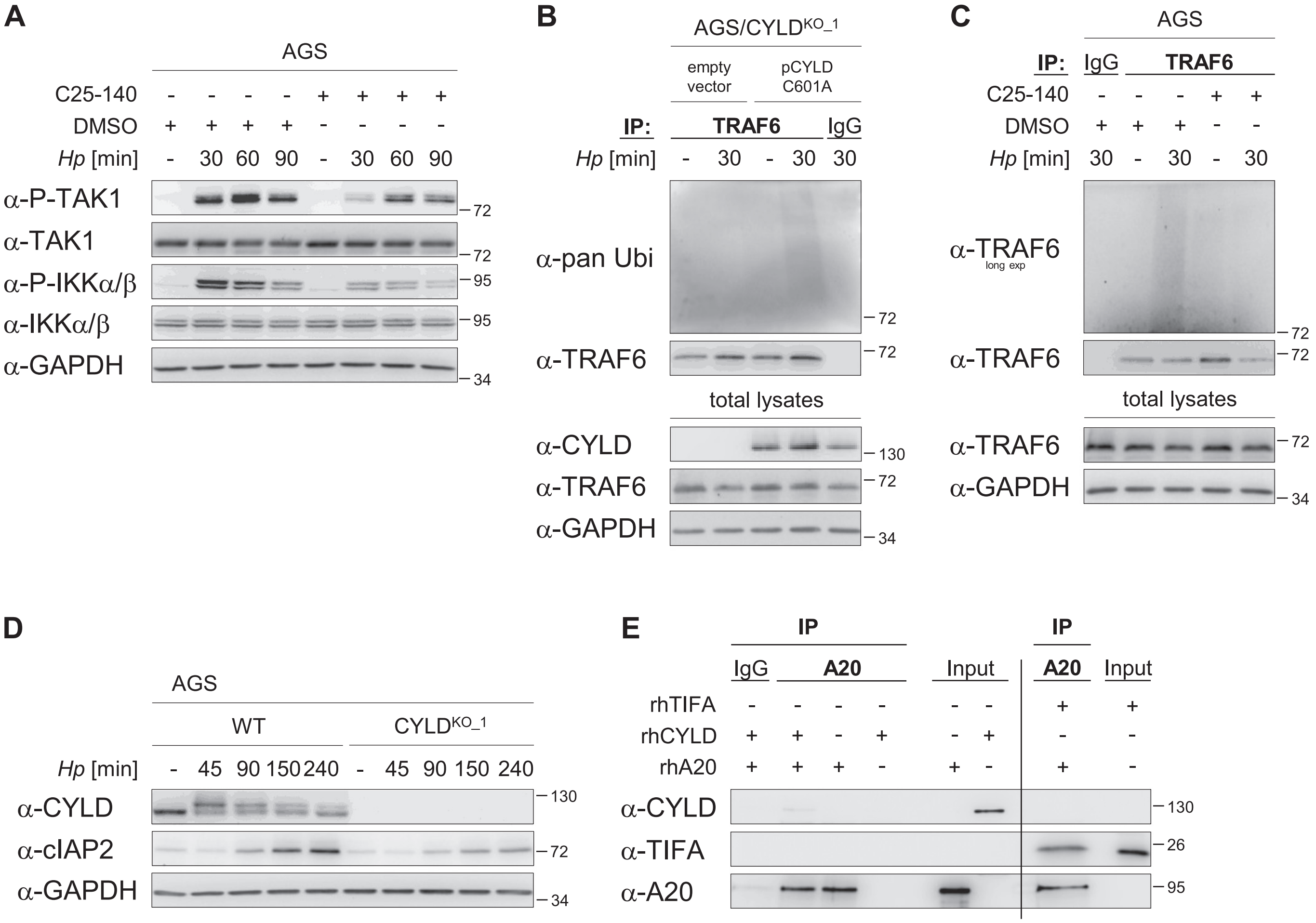

Figure EV3. CYLD stabilizes TRAF6 ubiquitinylation.

(**A**) AGS cells were incubated with DMSO (vehicle) or a selective TRAF6-Ubc13 inhibitor (C25-140, 20 μM) for 6 h followed by *H. pylori* infection for the times shown. Total lysates were analyzed by immunoblotting using the indicated antibodies. (**B**) $CYLD^{KO\_1}$ AGS cells were transfected with empty vector or plasmid expressing the catalytically inactive CYLD mutant (pCYLD C601A) 48 h prior to infection with *H. pylori* for 30 min. Total lysates were harvested using lysis buffer containing 1% SDS (denaturing condition). IP was performed using an anti-TRAF6 antibody or isotype-matched antibody (IgG). Eluates and total lysates were analyzed by immunoblotting using the indicated antibodies. (**C**) AGS cells were incubated with DMSO (vehicle) or a selective TRAF6-Ubc13 inhibitor (C25-140, 20 μM) for 6 h followed by *H. pylori* infection for 30 min. Total lysates were harvested using lysis buffer containing 1% SDS (denaturing condition). IP was performed using an anti-TRAF6 antibody or isotype-matched antibody (IgG). Eluates and total lysates were analyzed by immunoblotting using the indicated antibodies. (**D**) AGS cells were left uninfected (-) or infected with *H. pylori* for the times shown. Total lysates were analyzed by immunoblotting using the indicated antibodies. (**E**) Following incubation of recombinant human (rh) CYLD and rhA20 proteins or rhTIFA and rhA20 in vitro, IP was performed using an antibody against A20. Eluates and input (10 ng rhCYLD, rhA20 or rhTIFA proteins) were analyzed by immunoblotting using the indicated antibodies. For (**A**–**D**), GAPDH serves as the loading control for the total lysates. IBs were processed in parallel and depict 1 representative of at least two independent experiments. Source data are available online for this figure.

 © The Author(s)

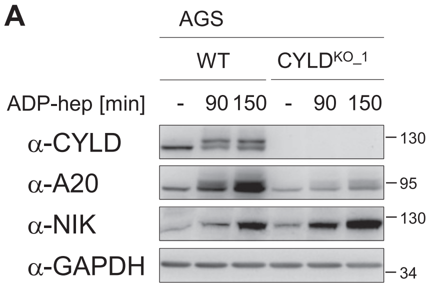

Figure EV4. CYLD negatively impacts *H. pylori*-induced alternative NF-κB activation through A20.

(**A**) WT and $CYLD^{KO\_1}$ AGS cells were left untreated (-) or treated with 200 nM ADP-heptose for the times shown. Total lysates were analyzed by immunoblotting using the indicated antibodies. (**B**) WT and $CYLD^{KO\_1}$ AGS cells were left untreated or treated with 30 ng/ml $LT\alpha_1\beta_2$ for the times shown. Total lysates were analyzed by immunoblotting using the indicated antibodies. For both panels, GAPDH serves as the loading control for the total lysates. IBs were processed in parallel and depict 1 representative of at least two independent experiments. Source data are available online for this figure.

© The Author(s)
